# Designing dicationic organic salts and ionic liquids exhibiting high fluorescence in the solid state

**DOI:** 10.1016/j.jil.2024.100125

**Published:** 2024-11-09

**Authors:** David King, Matthew C. Le, Yan P. Arnaiz, Seonghyeok L. Cox, Jakob Smith, Haesook Han, Pradip K. Bhowmik

**Affiliations:** Department of Chemistry and Biochemistry, University of Nevada Las Vegas, 4505 S. Maryland Parkway, Box 454003, Las Vegas, NV 89154, United States

**Keywords:** Synthesis, Structural design, Organic salts, Fluorescence, Solid state fluorescence, Optoelectronics

## Abstract

Dicationic ionic liquids (DILs) are emerging as a powerful, next-generation approach to designing applied ILs because of their superior physicochemical properties as well as their diverse complexity and tunability for task specific applications. DILs are scarce in the literature compared to monocationic ILs (MILs), and one of their main issues is their expected tendency to possess higher melting temperatures. A series of 1,4-bis[2-(4-pyridyl)ethenyl] benzene and 1,4-bis[2-(2-pyridyl)ethenyl]benzene quaternary salts (Q-BPEBs) with different counterions (bromide, tosylate, and triflimide) and carbon chain lengths (C_6_, C_9_, and C_12_) have been synthesized for their potential as DILs with strong photoluminescent properties in the solid state. All Q-BPEB salts demonstrated robust thermal stabilities as determined by thermogravimetric analysis (TGA). The differential scanning calorimetry (DSC) thermograms for Q-BPEB tosylates and triflimides displayed crystalline polymorphisms before melting transitions as verified by polarizing optical microscopy (POM). The Q-BPEB bromide and tosylate salts all showed high melting points of above >170 °C because of their dicationic rigid structures and strong ionic interactions of their anions. Once the Q-BPEB tosylates were exchanged with triflimide ions, *para*- isomers **1aTf**_**2**_**N, 1bTf**_**2**_**N**, and **1cTf**_**2**_**N** still possessed very high melting points (>225 °C), however, the *ortho*- isomers **2aTf**_**2**_**N, 2bTf**_**2**_**N**, and **2cTf**_**2**_**N** exhibited melting points lower than 100 °C, classifying them as DILs. Their photoluminescent properties were also studied in methanol with the emission values of λ_em_ = 476–482 nm for the *para*- isomers and those of λ_em_ = 448–453 nm for the *ortho*- isomers. In the solid state, the Q-BPEB salts exhibited strong fluorescence with quantum yields of up to 50 %. The relatively simple synthesis of these fluorescent dicationic organic salts and ILs are pertinent towards the scarcity of these materials in the literature and provide a deeper insight on the design of fluorescent ILs containing more than one charge center.

## 1. Introduction

Ionic liquids (ILs) are materials, typically organic ionic compounds, that possess melting points below 100 °C and have been a field of intense research in the past few decades. Monocationic ILs (MILs) are the most classical examples of ILs, which are simply composed of a cation/anion pair. Currently, MILs dominate the literature and have been extensively researched compared to ILs with more than one charge center. Dicationic ILs (DILs), which typically contain two cationic centers on the same molecular backbone with two counteranions, provide superior thermal stability and a higher degree of structural complexity to allow for the design of task-specific ILs (Shirota et al., 2011; Pandolfi et al., 2022). Additionally, DILs have been shown to be considerably less ecotoxic or even environmentally benign compared to their MIL counterparts and can be used as lipid analogous structures for lipid nanoparticle drug delivery systems (Montalbán et al., 2018; Kaczmarek et al., 2024; Kaur et al., 2024). The two cationic centers of a DIL are usually connected with an aliphatic or aromatic linker and can be fine-tuned by manipulations of the linker, sidechains, or even producing structurally complex asymmetric DILs (Zhang et al., 2008; Yu et al., 2007). An important consideration in the design of a DIL is to ensure that the structure contains proper functional groups that effectively lower the melting point of the DIL since the introduction of a second charge center increases stronger ionic interactions, which will result in an increase in the melting point of the material (Sun et al., 2018).

1,4-bis[2-(4-pyridyl)ethenyl]benzene and 1,4-bis[2-(2-pyridyl) ethenyl]benzene quaternary salts (Q-BPEBs), a class of dicationic organic salts, are bispyridinium salts extended with a double bond spacer from a core aromatic ring. Because of the extensive conjugation Q-BPEBs exhibit, they possess interesting photoluminescence properties in solution and crystalline states, including two and three-photon absorption (Gutov et al., 2011; Gao et al., 2016; Zhan et al., 2002). A major advantage of Q-BPEB is for designing task-specific materials. Q-BPEB is versatile in tunability as the N-linker can represent carbon chains of different lengths or functional groups, and the counteranion can be exchanged through simple metathesis reactions (Ni et al., 2016). Q-BPEB bromides of different carbon lengths are known to possess different aggregation behaviors for hydrogelator design, and counterion exchanges can result in augmented solubilities for aqueous systems (Bhattacharya and Samanta, 2012). Q-BPEB methyl iodide (Tang et al., 2003) and a Q-BPEB-dicucurbit[8]uril host-guest complex (Ni et al., 2016) are known to be water soluble which allows for biological imaging and chemical sensing (Zhou et al., 2019). Since their discovery, Q-BPEBs have found application as a fluorescent mitochondrial imaging probe (Chu et al., 2022), a photoisomerizable two-photon fluorescent DNA sequence-dependent imaging probe (Botti et al., 2019; Juskowiak, 2003), ratiometric heparin detection through multi-color emission switching (Dey et al., 2017), salt-induced color switching hydrogelators (Bhattacharya and Samanta, 2012), and construction of fluorescent host-guest supramolecular assemblies (Wu et al., 2020; Zhang et al., 2021).

Although some ILs contain aromatic/conjugated systems, such as imidazolium ILs, their photoluminescent properties are generally weak in solution (Paul et al., 2005). To augment the fluorescent properties of these weakly fluorescent ILs, the anion is selected from anionic fluorophores to fabricate an IL with integrated photochromic and photoluminescent properties (Zhang et al., 2011; Branco and Pina, 2009). ILs with intrinsic photoluminescent properties where the anion does not play an instrumental role in the photoluminescence have also been characterized, such as fluorescent imidazopyridine (Kitaoka et al., 2024) and quinolizinium ILs (Chen et al., 2011). It is an important consideration that if the cationic moiety of the IL itself is fluorescent, the anion can be freely exchanged with little effect to the photoluminescence to preserve opportunities for task-specific functionalities that the anion would introduce. Fluorescent ILs find pertinent applications in physical and chemical sensing (Ilyas et al., 2024), such as in the sensing of gaseous SO_2_ (Che et al., 2022). Fluorescent DILs are currently rare in the literature compared to MILs and need to be further characterized.

A significant roadblock of synthesizing Q-BPEBs is cumbersome synthesis and/or low yields. Current routes to synthesize Q-BPEBs either utilize the well-known Knoevenagel condensation from α/γ-picolinium salts and terepthalaldehyde using a basic catalyst, commonly piperidine (Tonga and Lahti, 2019; Gao et al., 2016; Yuan et al., 2007). In the other method of synthesis, non-quaternized BPEB is synthesized as a precursor, which uses expensive metal-containing catalysts (Gutov et al., 2009; Niknam et al., 2021; Li et al., 2010), harsh reaction conditions (Liu et al., 2019; Bhowmik et al., 2007), and caustic bases (Nabuurs et al. 2016; Detert et al., 2004). Q-BPEBs are typically paired with a halide counterion because of the alkylation reaction that utilizes alkyl halides, however other counterions paired with Q-BPEBs have been seldom researched. Additionally, there are no reports in the literature for Q-BPEPs as DILs since they are highly rigid rod-like molecules which render them very high melting points as well as strong ionic interactions. Both of these properties are not conducive for the preparation of ILs.

In this work, a facile synthesis for *ortho*- and *para*- Q-BPEB bromide salts with different carbon chain lengths (C_6_, C_9_, and C_12_) have been devised in fair to very good yields. The *ortho*- variant of Q-BPEB is scarce in the literature and has not been well characterized. Q-BPEB tosylates and triflimides were also prepared in fair to excellent yields for their potential to lower their melting points and become DILs. Their thermal properties as well as their photoluminescent properties in the solid and solution-state have been investigated herein. This route provides for chromatography-free purification for fluorescent dicationic styryl pyridinium salts and ILs in higher yields than previously reported procedures in the literature (Tonga and Lahti, 2019; Gao et al., 2016; Yuan et al., 2007).

Scheme 1, 2, 3

## 2. Materials and methods

### 2.1. General information

All chemicals and solvents were reagent grade and purchased from commercial vendors (Acros Organics, Alfa-Aesar, Sigma-Aldrich, and TCI America) and were used as received. The ^1^H and ^13^C nuclear magnetic resonance (NMR) salt solutions of the Q-BPEB salts were prepared by dissolving 10 mg of each of the salts in 1 mL DMSO-*d*_6_, and the spectra were recorded by using VNMR 400 spectrometer operating at 400 and 100 MHz, respectively, at room temperature and chemical shifts were referenced to tetramethylsilane (TMS) for ^1^H and ^13^C nuclei. Elemental analyses were performed by Atlantic Microlab Inc., Norcross, GA.

### 2.2. Synthesis

#### 2.2.1. General procedure for the synthesis of 1a-1c and 2a-2c alkylpicolinium bromide salts

The starting alkylpicolinium bromide salts were synthesized according to the literature procedure (Tonga and Lahti, 2019). **2b** was prepared by refluxing 3.56 g (38.28 mmol) of α-picoline with 9.52 g (47.33 mmol) 1-bromononane in 10 mL acetonitrile for 24 h The solvent was completely removed by a rotary evaporator and the resulting compound was washed with hexane to furnish 10.09 g of **2b** (88 %).

**2b**. (white solid). ^1^H NMR (400 MHz, CD_3_OD) *δ*=8.92 (d, *J* = 5.6 Hz, 1H), 8.45 (t, *J* =8.0 Hz, 1H), 8.01 (d, *J* =5.6 Hz, 1H), 7.94 (t, *J* =8.0 Hz, 1H), 4.61 (t, *J* = 8.0 Hz, 2H), 2.90 (s, 3H), 1.97 (m, 2H), 1.47 (m, 13H), 0.91 (t, *J* = 6.8 Hz, 3H). ^13^C NMR (100 MHz, CD_3_OD) *δ*=145.01, 144.96, 130.06, 125.54, 57.88, 31.54, 29.81, 29.06, 28.87, 28.75, 25.95, 22.26, 18.77, 12.97.

#### 2.2.2. General procedure for the synthesis of Q-BPEB bromide salts 1a-1cBr and 2a-2cBr

3.70 g (14.33 mmol) of **1a** was combined with 0.769 g (5.73 mmol) of terephthalaldehyde and 24 drops of piperidine in 50 mL of chloroform. After 48 h, the yellow precipitate was filtered off and washed with hot chloroform. For further purification, product **1aBr** was recrystallized from methanol for a final yield of 3.076 g (87 %) and stored away from light.

**1aBr**. (orange solid). ^1^H NMR (400 MHz, DMSO-*d*_6_) *δ*=8.95 (d, *J* = 4.8 Hz, 4H), 8.23 (d, *J* = 6.4 Hz, 4H), 8.04 (d, *J* = 16.4 Hz, 2H), 7.83 (s, 4H), 7.62 (d, *J* = 16.4 Hz, 2H), 4.48 (t, *J* = 7.2 Hz, 4H), 1.88 (m, 4H), 1.24 (m, 12H), 0.81 (m, 6H). Due to low solubility, the ^13^C NMR spectrum could not be acquired.

#### 2.2.3. Synthesis of Q-BPEB tosylate salts 1a-1cOTs and 2a-2cOTs

0.919 g (1.50 mmol) **1aBr** was dissolved in a 60 mL of hot methanol. 1.04 g (3.74 mmol) of silver tosylate was dissolved in minimum hot water and added to the flask covered in foil to protect it from light. After refluxing for 48 h, the silver bromide was filtered off and solvent was removed from the filtrate by a rotary evaporator. The yellow solid was dried in vacuo to remove any residual methanol. The solid was then washed with acetonitrile and syringe filtered after dissolving in methanol to remove any residual silver, furnishing 0.932 g (78 %) of **1aOTs**.

**1aOTs**. (orange solid). ^1^H NMR (400 MHz, DMSO-*d*_6_) *δ*=8.94 (d, *J* = 6.8 Hz, 4H), 8.22 (d, *J* = 6.8 Hz, 4H), 8.03 (d, *J* = 16.4 Hz, 2H), 7.82 (s, 4H), 7.61 (d, *J* = 16.4 Hz, 2H), 7.44 (d, *J* = 8.0 Hz, 4H), 7.06 (d, *J* = 7.6 Hz, 4H), 4.47 (t, *J* = 7.6 Hz, 4H), 2.23 (s, 6H), 1.87 (m, 4H), 1.24 (m, 12H), 0.81 (t, *J* =7.0 Hz, 6H). ^13^C NMR (100 MHz, DMSO-*d*_6_) *δ*=152.91, 146.24, 144.78, 140.18, 137.94, 137.33, 129.21, 128.43, 125.89, 124.47, 60.24, 30.98, 25.50, 22.28, 21.18, 14.24. Anal. Calc for C_46_H_56_N_2_O_6_S_2_ (797.08 g/mol): C, 69.31; H, 7.08; N, 3.51; S, 8.05 %. Found C, 69.05; H, 7.31; N, 3.63; S, 7.78 %.

#### 2.2.4. Synthesis of Q-BPEB triflimide salts 1a-1cTf_2_N and 2a-2cTf_2_N

1.03 g (1.29 mmol) of **1aOTs** was dissolved in 60 mL of hot methanol. 0.891 g (3.10 mmol) of lithium triflimide was added to the flask and brough to reflux. After 48 h, the solvent was removed by a rotary evaporator and washed with water. The solid was dissolved in warm acetonitrile, filtered, and the solvent was removed to furnish 1.25 g (95 %) of **1aTf**_**2**_**N**.

**1aTf**_**2**_**N**. (yellow solid). ^1^H NMR (400 MHz, DMSO-*d*_6_) *δ*=8.97 (d, *J* = 6.8 Hz, 4H), 8.24 (d, *J* = 6.8 Hz, 4H), 8.09 (d, *J* = 16.4 Hz, 2H), 7.85 (s, 4H), 7.63 (d, *J* = 16.4 Hz, 2H), 4.48 (t, *J* = 7.2 Hz, 4H), 1.89 (m, 4H), 1.27 (m, 12H), 0.84 (t, *J* = 7.2 Hz, 6H). ^13^C NMR (100 MHz, DMSO-*d*_6_) *δ*=152.94, 144.80, 140.20, 137.36, 129.23, 124.98, 124.49, 121.51, 118.31, 60.29, 31.00, 25.53, 22.30, 14.25. Anal. Calc for C_36_H_42_F_12_N_4_O_8_S_4_ (1014.98 g/mol): C, 42.60; H, 4.17; N, 5.52; S, 12.64 %. Found C, 42.90; H, 4.13; N, 5.53; S, 12.80 %.

### 2.3. Characterization techniques

The thermal stability properties of the salts were assessed using a TGA Q50 instrument at a heating rate of 10 °C⋅min^−1^ in nitrogen. The phase transition temperatures of the salts were determined using a TA module DSC Q200 series in nitrogen, at heating and cooling rates of 10 °C⋅min^−1^. The temperature axis of the DSC thermograms was calibrated with reference standards of high purity indium and tin. To verify phase transition data gathered from DSC, the crystalline polymorphisms of these salts were examined using a polarized optical microscopy (POM, Nikon, Model Labophot 2) equipped with crossed polarizers. The UV–Vis absorption spectra of the salts in spectrophotometric grade methanol were recorded by using the absorbance module attached to PerkinElmer Fluorescence Spectrometer FL 6500. The fluorescence and excitation spectra with the use of the single-cell holder accessory, as well as solution state absolute quantum yields with the use of an integrating sphere accessory, were measured with the same spectrometer. Absolute quantum yields of the salts in the solid state were measured with a Horiba Fluorolog fluorimeter (HORIBA Instruments Inc.) also equipped with an integrating sphere.

## 3. Results and discussion

### 3.1. Synthesis of dicationic ortho-/para- Q-BPEB bromides, tosylates, and triflimides

Q-BPEB bromides were successfully synthesized by Knoevenagel condensation through heating terephthalaldehyde and an excess of a α/γ-alkylpicolinium bromide salt with piperidine as a basic catalyst in chloroform followed by a simple workup. To furnish the Q-BPEB tosylates, the Q-BPEB bromides were refluxed with silver tosylate in methanol via metathesis reaction. Finally, to make the Q-BPEB triflimides, Q-BPEB tosylates were heated with lithium triflimide in methanol. The loss of the aromatic hydrogen peaks and benzylic methyl peak from the tosylate anion on the ^1^H NMR spectrum signified complete metathesis to triflimide anions. The purity of all Q-BPEB salts synthesized were determined by ^1^H and ^13^C NMR spectra (Figures S2–S19) and elemental analysis.

A significant advantage in the synthesis of Q-BPEBs utilizing this method is the versatile tunability of functional groups in each step of the synthesis. For the synthesis of α/γ-alkylpicolinium salts, carbon chains of different lengths and functionalities can be introduced when alkylated to picoline to yield a variety of α/γ-alkylpicolinium salts. Terephthalaldehyde containing functional groups on the aromatic ring can also be used to manipulate the electronic structure of the final Q-BPEB, which will also alter the melting point of the resulting compound depending on functionalization (Tonga and Lahti, 2019). Finally, metathesis reactions with different counteranions will yield new Q-BPEBs with impacted physical properties. The promotion of structural complexity for this class of dicationic organic molecules renders them prospective for task-specific IL applications due to the fine tunability of the physical and electronic structure. The cost of materials utilized in the synthesis are on par with common imidazolium/pyridinium ILs and are further mitigated due to the simple purification of Q-BPEBs.

### 3.2. Thermal stability

**Fig. 1.**
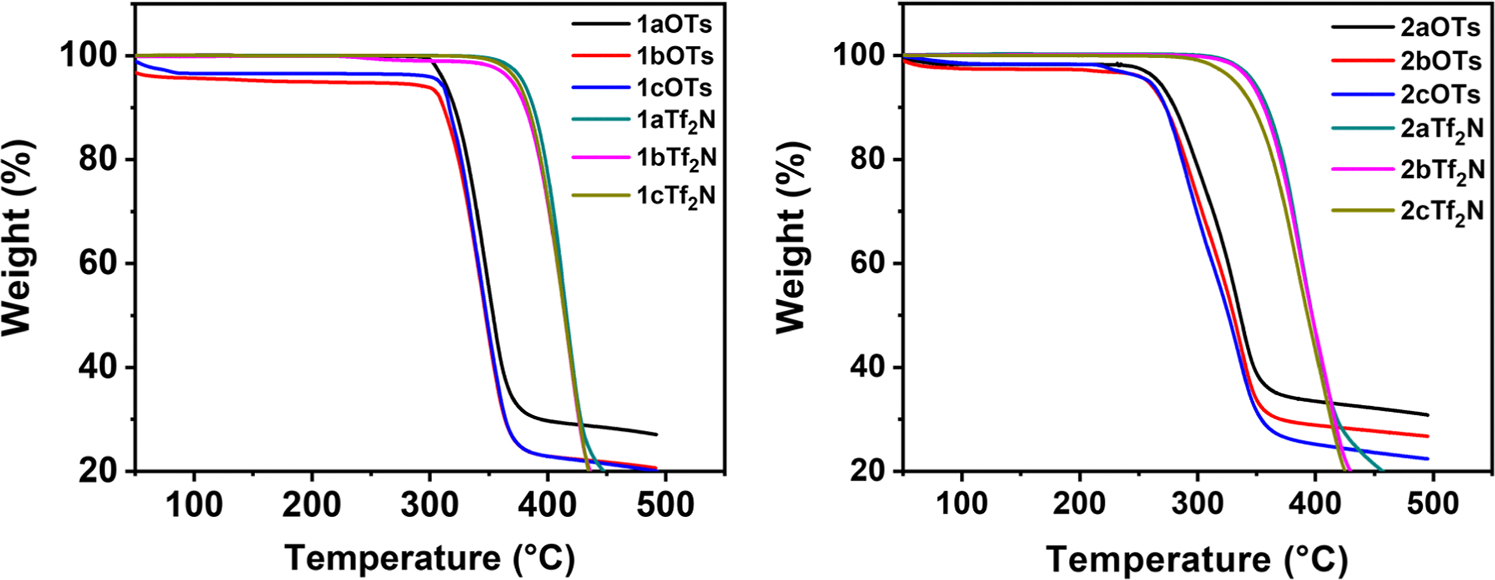
TGA thermograms of **1a-1cOTs** and **1a-1cTf**_**2**_**N** (left) and **2a-2cOTs** and **2a-2cTf**_**2**_**N** (right) obtained at heating rate of 10 °C/min in nitrogen.

The thermal stability for the *ortho*- and *para*- Q-BPEB bromides, tosylates, and triflimides were assessed by thermogravimetric analysis (TGA) and is defined as the temperature (°C) at which a 5 % weight loss occurred at a heating rate of 10 °C/min in nitrogen. The Q-BPEB bromides decomposed before their melting points in the range of 280–325 °C for the *para*- isomer and 232–262 °C for the *ortho*- isomer. The Q-BPEB tosylates and triflimides possessed considerably high thermal stabilities. For Q-BPEB triflimides, their thermal stabilities are comparable to imidazolium (Nirmale et al., 2022) and pyridinium triflimide DILs as reported in the literature (Mahrova et al., 2015). The thermal stabilities of *para*- Q-BPEBs **1a-1cOTs** were in the temperature range of 300–312 °C and those of **1a-1cTf2N** were in the range of 370–380 °C, displaying higher thermal stabilities due to the extraordinarily stable nature of the triflimide counterion (Bhowmik et al., 2020). On the other hand, *ortho*- Q-BPEBs were less thermally stable than their *para*- counterparts with **2a-2cOTs** possessing a temperature range of 257–267 °C and **2a-2cTf**_**2**_**N** with a range of 331–347 °C, respectively (Fig. 1).

### 3.3. Thermal properties

**Fig. 2.**
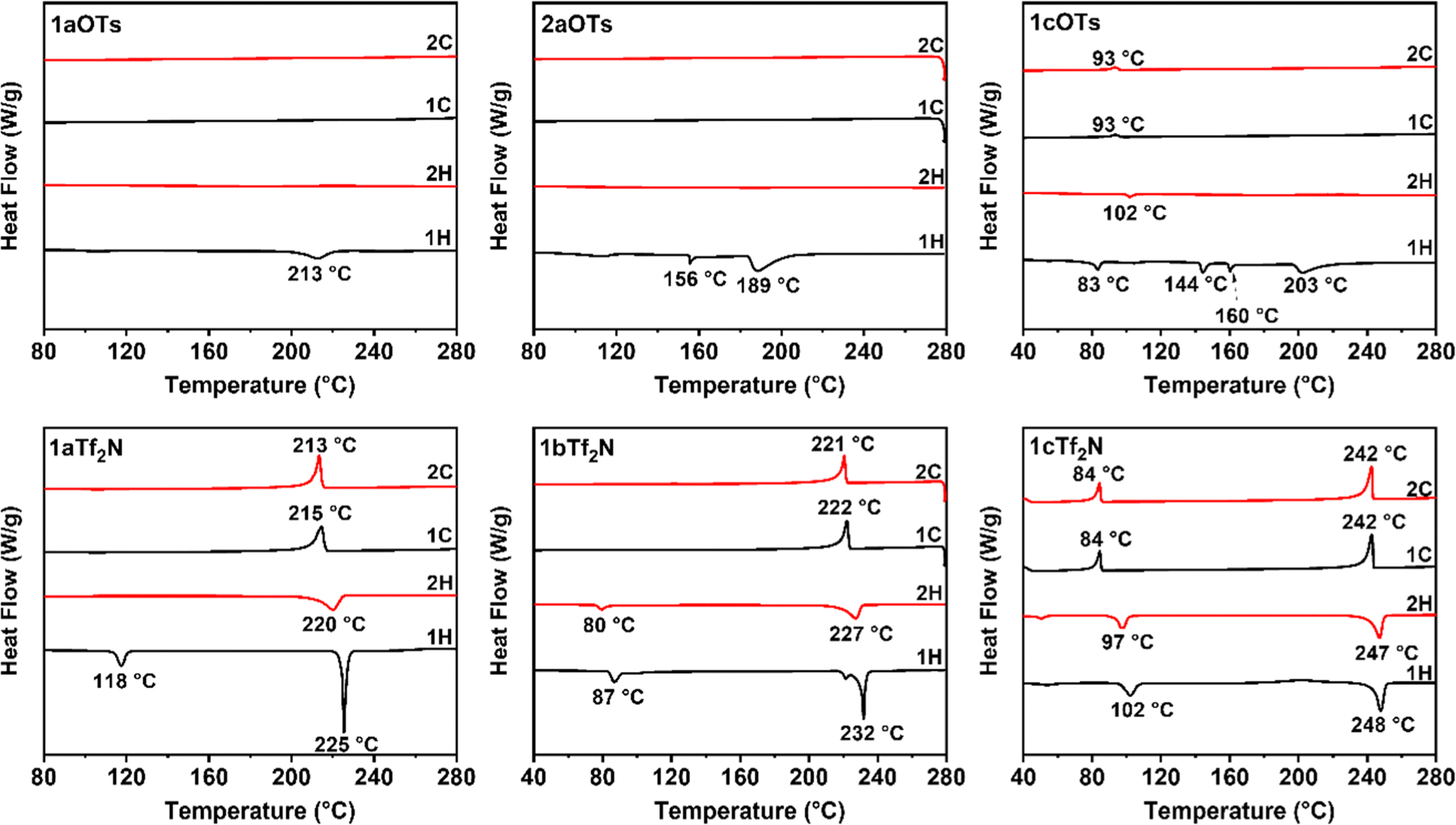
DSC thermograms of **1a-1cOTs** and **1a-1cTf**_**2**_**N** obtained at both heating and cooling rates of 10 °C⋅min^−1^ in nitrogen.

**Fig. 3.**
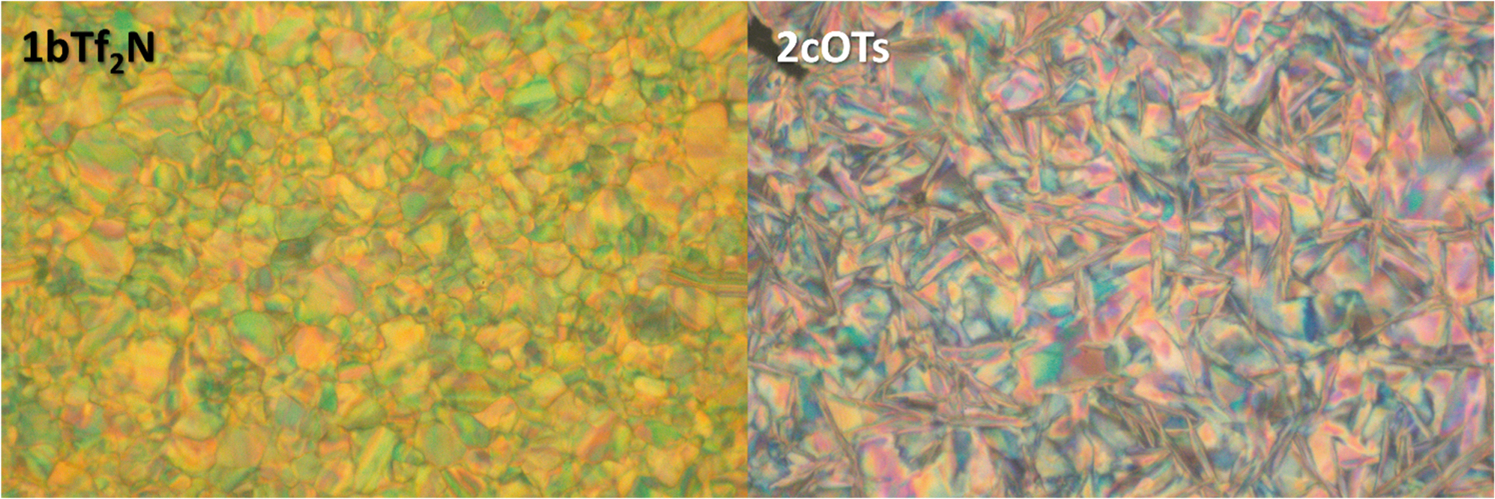
Photomicrographs of **1bTf**_**2**_**N** (left) acquired at 87 °C and **2cOTs** (right) acquired at 138 °C under crossed polarizers exhibiting birefringent crystal phases at 400x magnification.

DSC thermograms of *ortho*- and *para*- Q-BPEB bromides, tosylates, and triflimides were obtained to determine their phase transitions and melting points if they fit under the category of an ionic liquid (mp = <100 °C). For all Q-BPEB bromide salts, no phase transitions were observed until decomposition except for **2bBr** and **2cBr**, which possessed transitions that did not correspond to melting at 119 and 126 °C respectively. For the *para*- Q-BPEB tosylates (Fig. 2, **1a-1cOTs**), melting points were observed to be at higher temperatures (189–213 °C) with **1bOTs** and **1cOTs** possessing small endotherms before melting that corresponded to crystal-to-crystal transitions that were verified by POM (Fig. 3). The *para*- Q-BPEB triflimides (**1a-1cTf**_**2**_**N**) all displayed small endotherms (87–118 °C) before sharp endotherms that corresponded to higher melting temperatures compared to the *para*- Q-BPEB tosylates (225–248 °C). The DSC thermograms of the *para*- Q-BPEB triflimides suggested that these salts were more crystalline than the tosylate series since the endotherms appeared in the first and second heating cycles. In the triflimide series, exotherms appeared for both the first and second cooling cycles. In the tosylate series, there were endotherm(s) that appeared only in the first heating cycles but not in the second heating cycles.

**Fig. 4.**
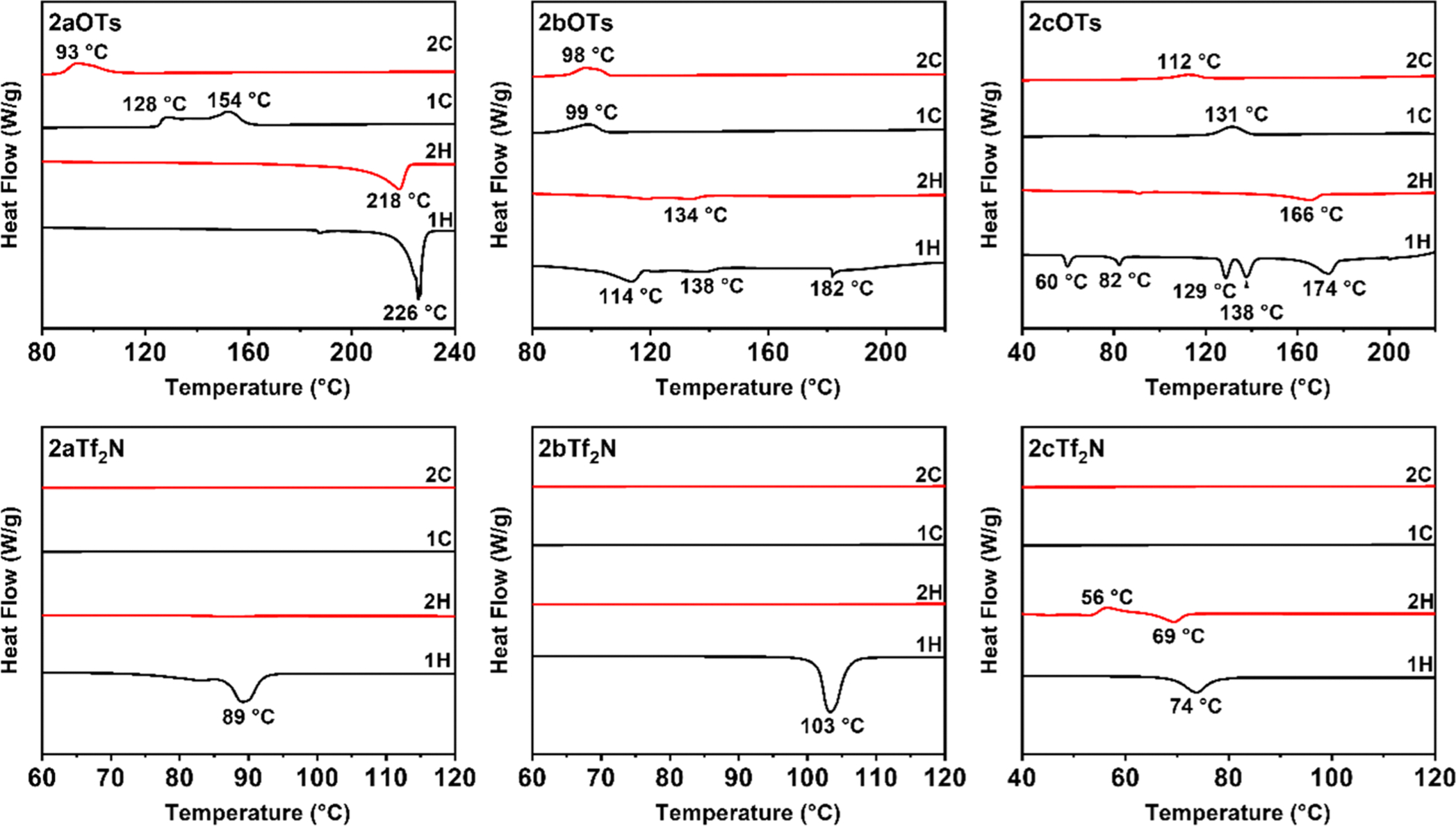
DSC thermograms of **2a-2cOTs** and **2a-2cTf**_**2**_**N** obtained at both heating and cooling rates of 10 °C⋅min^−1^ in nitrogen.

The *ortho*- Q-BPEB tosylates shared similar melting points to the *para*- isomers, however their melting points were more variable between the differing carbon-chain lengths (Fig. 4). While **2aOTs** had a sharp melting transition at 226 °C, **2bOTs** melted at 182 °C after two endotherms at 114 °C and 138 °C that corresponded to crystal-to-crystal transitions. For **2cOTs**, there were several defined endotherms at 60, 82, 129, and 138 °C that also corresponded to crystal-to-crystal transitions before melting at 174 °C. Considering the information gathered for the *ortho*- and *para*- Q-BPEB tosylates, there was not a significant change in their melting points between the structural isomers, which resulted in the tosylate series not to be ILs.

However, it was observed that the melting points of all *ortho*- Q-BPEB triflimides significantly decreased compared to their *para*- isomers. These results are expected as the *para*- isomers possess greater symmetry and stronger intermolecular interactions that increase the melting points of the materials. For **2aTf**_**2**_**N**, melting occurred at 89 °C which is about a 2.5 fold decrease in the melting point compared to the *para*- isomer (**1aTf**_**2**_**N**, 225 °C). **2cTf**_**2**_**N** displayed a melting point lower at 74 °C, about a 3-fold decrease in melting point compared to the *para*- isomer (**1cTf**_**2**_**N**, 248 °C). On the other hand, **2bTf**_**2**_**N** which contains the nine-carbon chain (C_9_) attached to the pyridinium *N*^+^ atom, had a slightly higher melting point at 103 °C than the two *ortho*- Q-BPEBs containing the six and twelve-carbon chain (C_6_ and C_12_). **2aTf**_**2**_**N, 2bTf**_**2**_**N**, and **2cTf**_**2**_**N** were successfully identified as DILs after properly manipulating the rigid structure by synthesizing the *ortho*- isomer containing triflimide anions. In the tosylate series, the endotherms appeared in the first heating cycles, but the endotherms in the second heating cycles became very weak, suggesting their lower crystallinity. In the triflimide series, the DSC thermograms displayed endotherms only in the first heating cycles, corresponding to their melting points. In subsequent heating cycles, there were no melting endotherms except for a small endotherm observed in **2cTf**_**2**_**N**. No exotherms were observed in any of their cooling cycles, suggesting that these salts were not capable of crystallizing after their melting points. These are inherent behaviors of true ILs. The combination of the *ortho*- structure and triflimide anions could be a good design strategy for the synthesis of novel DILs.

### 3.4. Optical properties

The optical properties of the *ortho*- and *para*- Q-BPEBs containing the C_6_ chain length with ^−^Br, ^−^OTs, and Tf_2_N^−^ counterions were studied in methanol at 1.0–5.0 × 10^−5^ M concentrations. Their molar absorptivities were also calculated and found to be in the range of 17,344–61,075 *M*^−1^ cm^−1^ (Table 1). The UV–Visible spectra (Figures S20–S22) for the *para*- isomers showed λ_abs_ peaks at λ_abs_ = 249–251 nm and λ_abs_ = 383–399 nm while the *ortho*- salts (Figure S23–S25) only had one λ_abs_ peaks at λ_abs_ = 382–384 nm. The *para*- Q-BPEB salts displayed their λ_ex_ peaks at 422–430 nm and their λ_em_ peaks were in the range of 476–482 nm. For the *ortho*- isomers, their λ_ex_ peaks were at 410/411 nm and their λ_em_ peaks were in the range of 448–453 nm. The solution state quantum yields for these salts were in the range of 5–10 %. Data for optical properties, absorption spectra, and photoluminescence spectra of the Q-BPEB salts containing C_9_ and C_12_ chain lengths are contained in the supplementary materials (Table S1 and Figures S26–S49).

Fig. 5

**Fig. 6.**
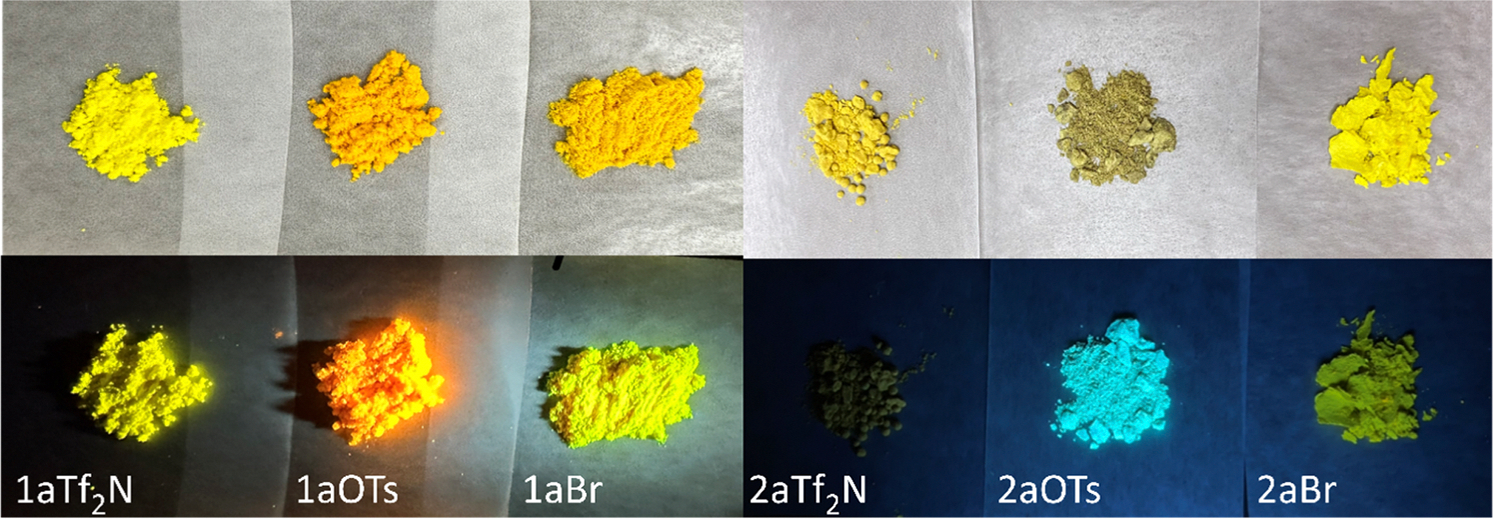
(a) Fluorescence of **1a** and **2a** Q-BPEB salts in the solid state using a handheld UV lamp (bottom) and appearance of those salts in ambient light (top).

As shown in Fig. 6, most of the Q-BPEB salts displayed little to strong fluorescence in the solid state, and their absolute quantum yields were measured. Overall, all Q-BPEB bromides had little to undetectable fluorescence, while the tosylate and triflimide Q-BPEBs possessed stronger fluorescence. The *para*- Q-BPEBs generally had higher quantum yields than the *ortho*- isomers with **1bTf**_**2**_**N** possessing the highest absolute quantum yield of 50 %. This phenomenon could be due to the difference in crystal packing between the *ortho*- and *para*- isomers in which the *para*- isomers possess a greater degree of aggregation-induced emission (AIE) in the solid state (Xu et al., 2020; Mei et al., 2015; Bhowmik et al., 2021). Generally, quantum yields are low in the solid state because of aggregation-induced quenching (AIQ), while quantum yields are high in solutions because aggregation phenomena do not occur due to dilution effect. Quantum yields of Q-BPEB bromide salts were 0–4 % due to AIQ in the solid state, but those of Q-BPEB tosylates and triflimides, with the exception two triflimide salts, underwent AIE in the solid state. AIE has been studied for many organic luminophores for the last two decades and has pinpointed the interest of Q-BPEB tosylate and Q-BPEB triflimide salts as promising materials for potential applications in optoelectronic technologies (Xu et al., 2020; Rodrigues and Seixas de Melo, 2021) Table 2.

## 4. Conclusion

A series of fluorescent Q-BPEB bromides, tosylates, and triflimides containing carbon chain lengths of C_6_, C_9_, or C_12_ were synthesized utilizing an improved synthesis and investigated for their potential to form DILs. Their purity was confirmed by ^1^H and ^13^C NMR as well as elemental analysis. Their thermal stabilities was measured by TGA and their phase transitions by DSC analysis. DSC thermograms confirmed that **2aTf**_**2**_**N, 2bTf**_**2**_**N**, and **2cTf**_**2**_**N** are DILs and that the *ortho*- isomer containing triflimide counterions is structurally necessary to synergistically lower the melting points of the dicationic salts. The photoluminescent properties were also characterized in the solution and solid state, demonstrating strong absorption and photoluminescent properties. Dicationic organic salts and ILs with diversified rigid organic structures were successfully synthesized, and insights can be used to design exciting new classes of DILs exhibiting strong photophysical properties for task-specific optoelectronic and sensing applications.

## Supplementary Material

1

## Figures and Tables

**Fig. 5.**
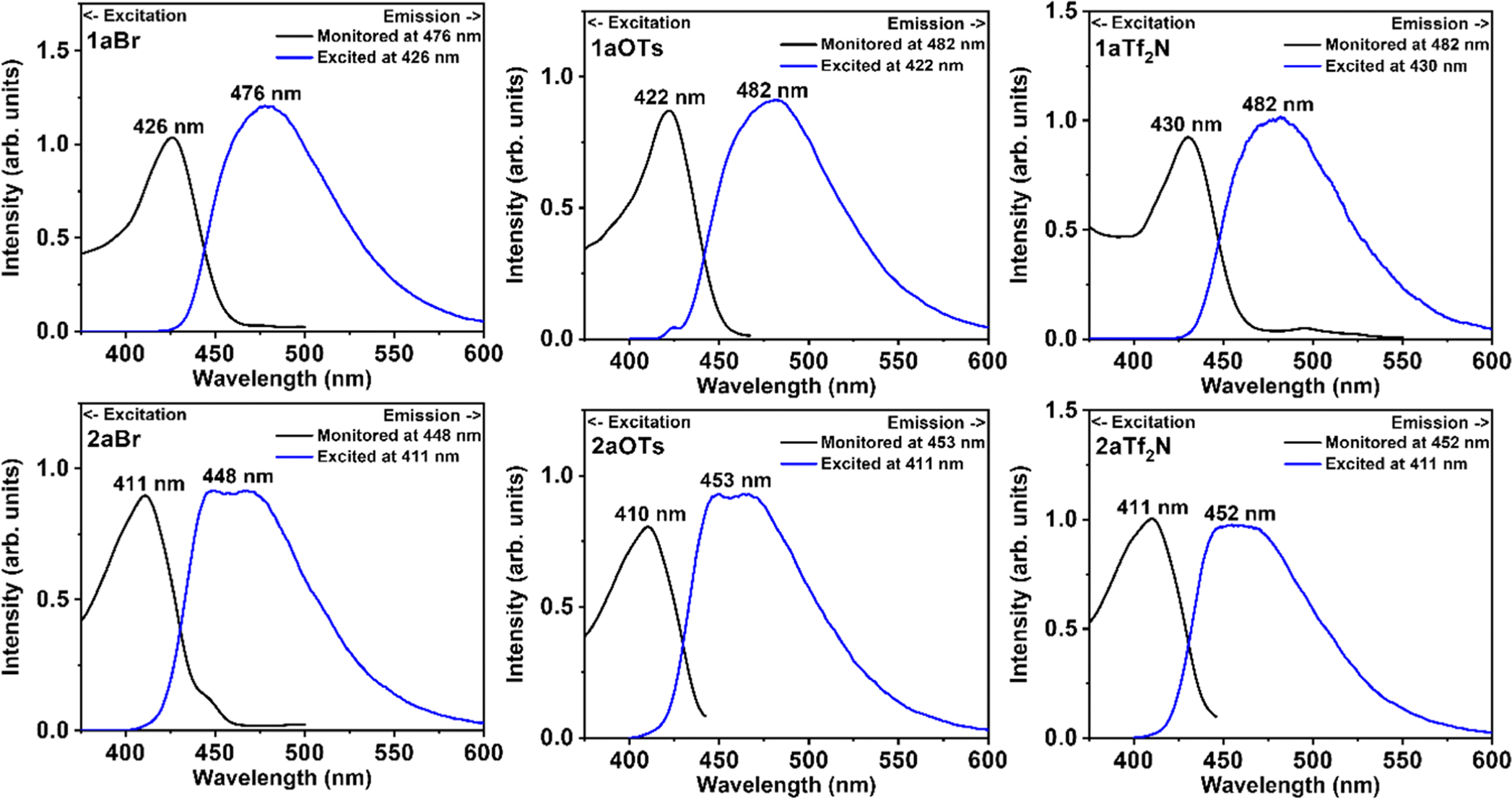
Room-temperature emission spectra (right) and excitation spectra (left) of **1aBr, 1aOTs**, and **1aTf**_**2**_**N** (top) and **2aBr, 2aOTs, 2aTf**_**2**_**N** (bottom) dissolved in methanol (concentration 5.0 × 10^−5^ M). The emission spectra were measured by exciting the solutions around the lowest-energy absorption peaks. Excitation spectra were recorded by monitoring the fluorescence around 478 nm or 451 nm as the wavelength of the excitation wavelength was varied.

**Scheme 1.**
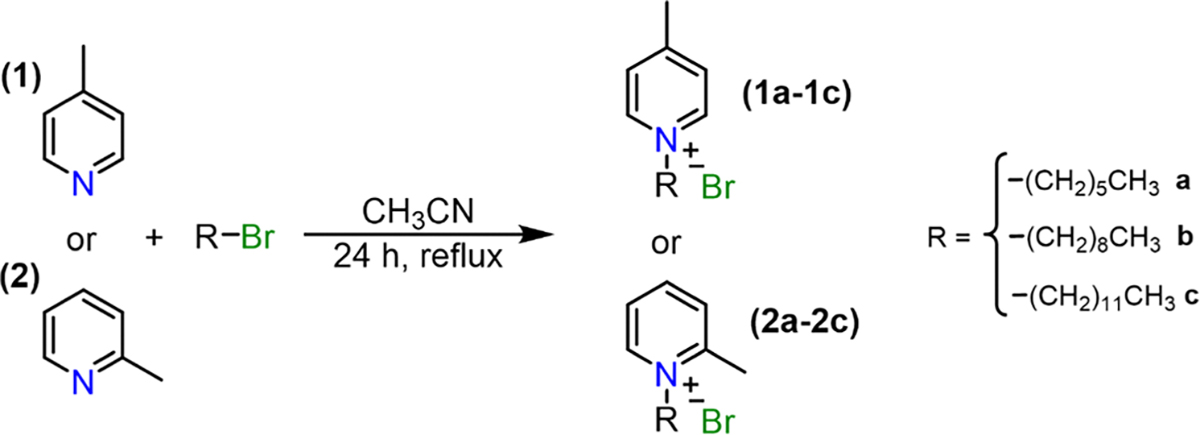
Synthesis of α/γ-alkylpicolinium bromide salts **1a-1c** and **2a-2c**.

**Scheme 2.**
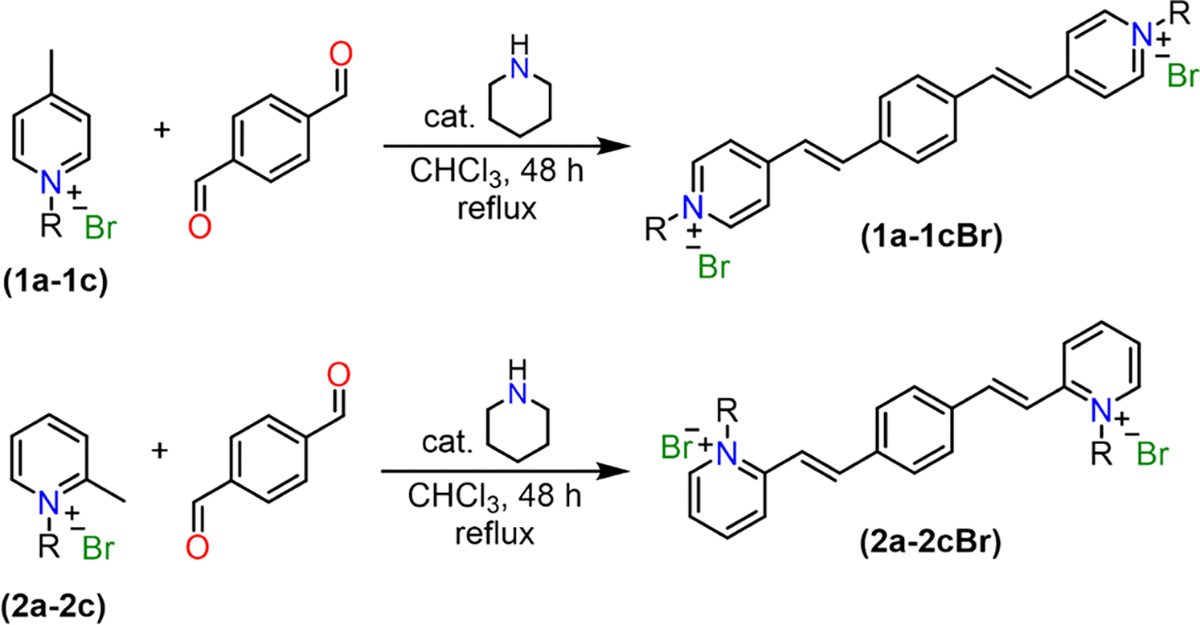
Synthesis of *ortho*- and *para*- Q-BPEB salts **1a-1cBr** and **2a-2cBr.**

**Scheme 3.**
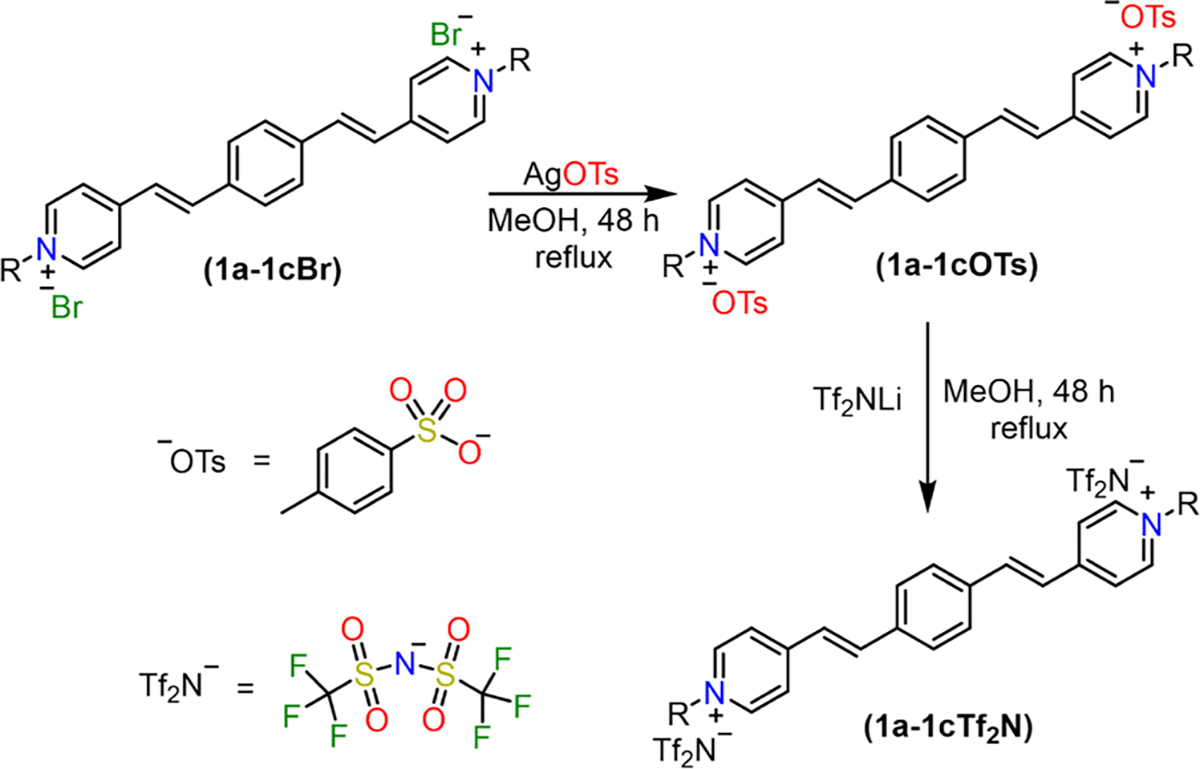
Synthesis of *para*- Q-BPEB tosylates and triflimides **1a-1cOTs** and **1a-1cTf**_**2**_**N** via metathesis reaction. *Ortho*- Q-BPEB salts **2a-2cOTs** and **2a-2cTf**_**2**_**N** were synthesized according to the identical procedures adopted for the *para*- isomers.

**Table 1:** Optical absorption and photoluminescent properties of C_6_ *ortho*- and *para*- Q- BPEBs.

Q-BPEB	λ_abs_ wavelength (nm)	Molar absorptivity (*M*^−1^ cm^−1^)	λ_em_ wavelength (nm)

**1aBr**	251	ε_251_ = 20,070 ± 204	476
	394	ε_394_ = 59,672 ± 2485	
**1aOTs**	251	ε_251_ = 21,786 ± 77	482
	383	ε_383_ = 45,803 ± 278	
**laTf_2_N**	249	ε_249_ = 17,344 ± 154	482
	399	ε_399_ = 61,075 ± 3899	
**2aBr**	384	ε_384_ = 55,071 ± 1195	448
**2aOTs**	384	ε_384_ = 41,131 ± 100	453
**2aTf_2_N**	382	ε_382_ = 54,974 ± 1681	452

**Table 2:** Solid-state quantum yields of Q-BPEB salts.

Q-BPEB	^-^Br	^-^OTs	Tf_2_N^-^

**1a**	4 %	24 %	12 %
**1b**	2 %	18 %	50 %
**1c**	3 %	23 %	5 %
**2a**	-	12 %	-
**2b**	3 %	5 %	-
**2c**	-	3 %	10 %

## Data Availability

Data will be made available on request.
