# Supplementary material for: Designing dicationic organic salts and ionic liquids exhibiting high fluorescence in the solid state
Source: J Ion Liq. Author manuscript; Available in PMC 2025 Jan 9. (PMC11711018; doi:10.1016/j.jil.2024.100125)
Supplement: 1 [file NIHMS2042941-supplement-1.docx]

# **Supplementary Material**

# Designing dicationic organic salts and ionic liquids exhibiting high fluorescence in the solid state

David King^a*^, Matthew C. Le^a^, Yan P. Arnaiz^a^, Seonghyeok L. Cox^a^, Jakob Smith^a^, Haesook Han^a^, Pradip K. Bhowmik^a^

*^a^ Department of Chemistry and Biochemistry,* University *of Nevada Las Vegas, 4505 S. Maryland Parkway, Box 454003, Las Vegas, NV 89154, USA*

| **NMR Information:** |  |  |
| --- | --- | --- |
| NMR Data and yields of Q-BPEB salts | Pages 4-7 |  |
| ^1^H and ^13^C NMR spectra of **2b** in CD_3_OD | Page 8 |  |
| ^1^H NMR spectrum of **1aBr** in *d*_6_-DMSO | Page 9 |  |
| ^1^H and ^13^C NMR spectra of **1bBr** in *d*_6_-DMSO | Page 10 |  |
| ^1^H and ^13^C NMR spectra of **1cBr** in *d*_6_-DMSO | Page 11 |  |
| ^1^H and ^13^C NMR spectra of **2aBr** in *d*_6_-DMSO | Page 12 |  |
| ^1^H and ^13^C NMR spectra of **2bBr** in *d*_6_-DMSO | Page 13 |  |
| ^1^H and ^13^C NMR spectra of 2cBr in *d*_6_-DMSO | Page 14 |  |
| ^1^H and ^13^C NMR spectra of **1aOTs** in *d*_6_-DMSO | | Page 15 |
| ^1^H and ^13^C NMR spectra of **1bOTs** in *d*_6_-DMSO | | Page 16 |
| ^1^H and ^13^C NMR spectra of **1cOTs** in *d*_6_-DMSO | | Page 17 |
| ^1^H and ^13^C NMR spectra of **2aOTs** in *d*_6_-DMSO | | Page 18 |
| ^1^H and ^13^C NMR spectra of **2bOTs** in *d*_6_-DMSO | | Page 19 |
| ^1^H and ^13^C NMR spectra of 2cOTs in *d*_6_-DMSO | | Page 20 |
| ^1^H and ^13^C NMR spectra of 1aTf_2_N in *d*_6_-DMSO | | Page 21 |
| ^1^H and ^13^C NMR spectra of 1bTf_2_N in *d*_6_-DMSO | | Page 22 |
| ^1^H and ^13^C NMR spectra of 1cTf_2_N in *d*_6_-DMSO | | Page 23 |
| ^1^H and ^13^C NMR spectra of 2aTf_2_N in *d*_6_-DMSO | | Page 24 |
| ^1^H and ^13^C NMR spectra of 2bTf_2_N in *d*_6_-DMSO | | Page 25 |
| ^1^H and ^13^C NMR spectra of 2cTf_2_N in *d*_6_-DMSO | | Page 26 |
| Optical Absorption and photoluminescent properties of C_9_ and C_12_ Q-BPEBs | | Page 27 |
| UV-Visible Absorption Spectra: | |  |
| 1aBr | | Page 28 |
| **1aOTs** | | Page 28 |
| **1aTf_2_N** | | Page 29 |
| **2aBr** | | Page 29 |
| **2aOTs** | | Page 30 |
| **2aTf_2_N** | | Page 30 |
| **1bBr** | | Page 31 |
| **1bOTs** | | Page 31 |
| **1bTf_2_N** | | Page 32 |
| **2bBr** | | Page 32 |
| **2bOTs** | | Page 33 |
| **2bTf_2_N** | | Page 33 |
| **1cBr** | | Page 34 |
| **1cOTs** | | Page 34 |
| **1cTf_2_N** | | Page 35 |
| **2cBr** | | Page 35 |
| **2cOTs** | | Page 36 |
| **2cTf_2_N** | | Page 36 |
| Emission/Excitation Spectra: | |  |
| 1bBr | | Page 37 |
| **1bOTs** | | Page 37 |
| **1bTf_2_N** | | Page 38 |
| **2bBr** | | Page 38 |
| **2bOTs** | | Page 39 |
| **2bTf_2_N** | | Page 39 |
| **1cBr** | | Page 40 |
| **1cOTs** | | Page 40 |
| **1cTf_2_N** | | Page 41 |
| **2cBr** | | Page 41 |
| **2cOTs** | | Page 42 |
| **2cTf_2_N** | | Page 42 |

**1bBr**. (orange solid, recrystallized from MeOH, 3.85 g, yield 77%). ^1^H NMR (400 MHz, DMSO-*d*_6_) *δ=*9.00 (d, J= 6.4 Hz, 4H), 8.28 (d, J= 6.4 Hz, 4H), 8.09 (d, J= 16.4 Hz, 2H), 7.86 (s, 4H), 7.66 (d, J= 16 Hz, 2H), 4.51 (t, J= 7.2 Hz, 4H), 1.89 (m, 4H), 1.26 (m, 24H), 0.83 (t, J= 7.2 Hz, 6H). ^13^C NMR (100 MHz, DMSO-*d*_6_) *δ*=152.96, 144.86, 144.81, 140.23, 137.38, 129.24, 124.50, 60.23, 31.65, 30.99, 29.18, 29.00, 28.84, 25.86, 22.51, 14.40.

**1cBr**. (orange solid, recrystallized from EtOH, 2.09 g, yield 67%). ^1^H NMR (400 MHz, DMSO-*d*_6_) *δ=*8.99 (d, J= 6.4 Hz, 4H), 8.28 (d, J= 6.4 Hz, 4H), 8.09 (d, J= 16 Hz, 2H), 7.86 (s, 4H), 7.66 (d, J= 16.4 Hz, 2H), 4.51 (t, J= 6.8 Hz, 4H), 1.88 (m, 4H), 1.25 (m, 36H), 0.83 (t, J= 6.0 Hz, 6H). ^13^C NMR (100 MHz, DMSO-*d*_6_) *δ*=152.95, 144.81, 140.22, 137.38, 129.24, 124.98, 124.48, 60.25, 31.72, 30.96, 29.43, 29.32, 29.20, 29.14, 28.81, 25.84, 22.52, 14.39.

**2aBr**. (yellow solid, recrystallized from EtOH, 1.58 g, yield 73%). ^1^H NMR (400 MHz, DMSO-*d*_6_) *δ=*9.02 (d, J= 6 Hz, 2H), 8.56 (m, 4H), 8.01 (m, 8H), 7.80 (d, J= 16 Hz, 2H), 4.86 (t, J= 7.6 Hz, 4H), 1.83 (m, 4H), 1.32 (m, 12H), 0.80 (t, J= 6.8 Hz, 6H). ^13^C NMR (100 MHz, DMSO-*d*_6_) *δ*=152.02, 146.15, 145.14, 142.67, 137.27, 129.60, 126.53, 126.28, 119.03, 58.12, 30.98, 30.22, 25.51, 22.29, 14.22.

**2bBr**. (yellow solid, recrystallized from EtOH, 3.52 g, yield 88%). ^1^H NMR (400 MHz, DMSO-*d*_6_) *δ=*9.03 (d, J= 7.2 Hz, 2H), 8.56 (m, 4H), 8.01 (m, 8H), 7.81 (d, J= 16 Hz, 2H), 4.86 (t, J= 7.2 Hz, 4H), 1.82 (m, 4H), 1.32 (m, 24H), 0.80 (t, J= 6 Hz, 6H). ^13^C NMR (100 MHz, DMSO-*d*_6_) *δ*=152.04, 146.17, 145.16, 142.65, 137.28, 129.59, 126.55, 126.30, 119.05, 58.09, 31.65, 30.24, 29.12, 28.96, 28.77, 25.78, 25.78, 22.48, 14.38.

**2cBr**. (yellow solid, recrystallized from EtOH, 4.31 g, yield 66%). ^1^H NMR (400 MHz, DMSO-*d*_6_) *δ=*9.02 (d, J= 6.4 Hz, 2H), 8.56 (d, 4H), 8.00 (m, 8H), 7.81 (d, J= 16 Hz, 2H), 4.86 (t, J= 7.2 Hz, 4H), 1.82 (m, 4H), 1.32 (m, 36H), 0.80 (t, J= 6.4 Hz, 6H). ^13^C NMR (100 MHz, DMSO-*d*_6_) *δ*=152.02, 146.89, 145.16, 142.66, 137.27, 129.58, 126.50, 126.31, 119.03, 58.07, 31.72, 30.25, 29.45, 29.41, 29.32, 29.16, 28.76, 25.77, 22.52, 14.38.

**1bOTs**. (orange solid 2.59 g, yield 81%). ^1^H NMR (400 MHz, DMSO-*d*_6_) *δ=*8.99 (d, J= 6.4 Hz, 4H), 8.27 (d, J= 6.8 Hz, 4H), 8.08 (d, J= 16.4 Hz, 2H), 7.87 (s, 4H), 7.66 (d, J= 16.4 Hz, 2H), 7.49 (d, J= 8.0 Hz, 4H), 7.12 (d, J= 7.6 Hz, 4H), 4.51 (t, J= 7.2 Hz, 4H), 2.28 (s, 6H), 1.92 (m, 4H), 1.25 (m, 24H), 0.86 (t, J= 7.0 Hz, 6H). ^13^C NMR (100 MHz, DMSO-*d*_6_) *δ*=152.92, 146.27, 144.80, 140.20, 137.95, 137.36, 129.23, 128.45, 125.91, 124.49, 60.27, 31.65, 30.98, 28.99, 25.83, 22.51, 21.20, 14.39. Anal. Calc for C_52_H_68_N_2_O_6_S_2_ (881.24 g/mol): C, 70.87; H, 7.78; N, 3.18; S, 7.28%. Found C, 70.20; H, 7.79; N, 3.16; S, 6.88%.

**1cOTs**. (orange solid 2.36 g, yield 64%). *δ=*8.93 (d, J= 6.8 Hz, 4H), 8.21 (d, J= 6.8 Hz, 4H), 8.02 (d, J= 16.4 Hz, 2H), 7.81 (s, 4H), 7.597 (d, J= 16.4 Hz, 2H), 7.43 (d, J= 8.4 Hz, 4H), 7.05 (d, J= 8.0 Hz, 4H), 4.46 (t, J= 7.2 Hz, 4H), 2.22 (s, 6H), 1.86 (m, 4H), 1.17 (m, 36H), 0.78 (t, J= 7.0 Hz, 6H). ^13^C NMR (100 MHz, DMSO-*d*_6_) *δ*=152.88, 146.24, 144.76, 140.15, 137.89, 137.31, 129.18, 128.39, 125.86, 124.93, 124.43, 60.23, 31.68, 30.91, 29.38, 29.09, 25.79, 22.48, 21.16, 14.34. Anal. Calc for C_58_H_80_N_2_O_6_S_2_ (965.41 g/mol): C, 72.16; H, 8.35; N, 2.90; S, 6.64%. Found C, 71.68; H, 8.31; N, 2.87; S, 6.41%.

**2aOTs**. (yellow solid 1.55 g, yield 65%). *δ=*8.98 (d, J= 8 Hz, 2H), 8.53 (d, J= 4.4 Hz, 4H), 7.97 (m, 8H), 7.76 (d, J= 16 Hz, 2H), 7.46 (d, J= 8 Hz, 4H), 7.08 (d, J= 8.0 Hz, 4H), 4.81 (t, J= 7.2 Hz, 4H), 2.25 (s, 6H), 1.81 (m, 4H), 1.31 (m, 12H), 0.82 (t, J= 7.2 Hz, 6H). ^13^C NMR (100 MHz, DMSO-*d*_6_) *δ*=151.98, 146.25, 146.13, 145.14, 142.64, 137.96, 137.25, 129.56, 128.45, 125.91, 118.98, 58.07, 30.97, 30.20, 25.51, 22.28, 21.20, 14.21. Anal. Calc for C_46_H_56_N_2_O_6_S_2_ (797.08 g/mol): C, 69.31; H, 7.08; N, 3.51; S, 8.05%. Found C, 69.43; H, 7.05; N, 3.56; S, 8.05%.

**2bOTs**. (yellowish-green solid 2.85 g, yield 89%). ^1^H NMR (400 MHz, DMSO-*d*_6_) *δ=*8.97 (d, J= 6 Hz, 2H), 8.52 (d, J= 4.4 Hz, 4H), 7.97 (m, 8H), 7.76 (d, J= 16 Hz, 2H), 7.46 (d, J= 8 Hz, 4H), 7.08 (d, J= 8.0 Hz, 4H), 4.81 (t, J= 7.2 Hz, 4H), 2.25 (s, 6H), 1.81 (m, 4H), 1.31 (m, 6H), 0.82 (t, J= 7.2 Hz, 6H). ^13^C NMR (100 MHz, DMSO-*d*_6_) *δ*=151.99, 146.23, 146.15, 145.14, 142.61, 137.97, 137.25, 129.54, 128.45, 126.52, 126.29, 125.91, 119.00, 58.05, 31.64, 30.20, 29.12, 28.94, 25.77, 22.47, 21.20, 14.36. Anal. Calc for C_52_H_68_N_2_O_6_S_2_ (881.24 g/mol): C, 70.87; H, 7.78; N, 3.18; S, 7.28%. Found C, 69.87; H, 7.81; N, 3.25; S, 7.00%.

**2cOTs**. (yellow solid 2.37 g, yield 79%). ^1^H NMR (400 MHz, DMSO-*d*_6_) *δ=*8.98 (d, J= 6.4 Hz, 2H), 8.53 (d, J= 4.4 Hz, 4H), 7.97 (m, 8H), 7.77 (d, J= 16 Hz, 2H), 7.46 (d, J= 8 Hz, 4H), 7.08 (d, J= 8 Hz, 4H), 4.81 (t, J= 7.2 Hz, 4H), 2.24 (s, 6H), 1.80 (m, 4H), 1.34 (m, 36H), 0.79 (t, J= 7.2 Hz, 6H). ^13^C NMR (100 MHz, DMSO-*d*_6_) *δ*=151.98, 146.25, 145.16, 145.15, 142.62, 137.95, 137.25, 129.54, 128.44, 125.91, 118.98, 58.03, 31.71, 30.22, 29.44, 29.40, 29.30, 29.14, 28.74, 25.76, 22.51, 21.20, 14.36. Anal. Calc for C_58_H_80_N_2_O_6_S_2_ (965.41 g/mol): C, 72.16; H, 8.35; N, 2.90; S, 6.64%. Found C, 72.13; H, 8.32; N, 2.91; S, 6.61%.

**1bTf_2_N**. (orange solid 1.16 g, yield 77%). ^1^H NMR (400 MHz, DMSO-*d*_6_) *δ=*8.95 (d, J= 6.8 Hz, 4H), 8.24 (d, J= 6.8 Hz, 4H), 8.04 (d, J= 16.4 Hz, 2H), 7.84 (s, 4H), 7.62 (d, J= 16.4 Hz, 2H), 4.49 (t, J= 7.6 Hz, 4H), 1.88 (m, 4H), 1.26 (m, 24H), 0.82 (t, J= 7.2 Hz, 6H). ^13^C NMR (100 MHz, DMSO-*d*_6_) *δ*=152.93, 144.80, 140.19, 137.35, 129.22, 124.97, 124.48, 121.50, 118.30, 60.29, 31.64, 30.97, 29.17, 28.98, 28.82, 25.86, 22.50, 14.38. Anal. Calc for C_42_H_54_F_12_N_4_O_8_S_4_ (1099.13 g/mol): C, 45.90; H, 4.95; N, 5.10; S, 11.67%. Found C, 46.08; H, 5.07; N, 5.14; S, 11.59%.

**1cTf_2_N**. (orange solid 0.726 g, yield 64%). ^1^H NMR (400 MHz, DMSO-*d*_6_) *δ=*8.96 (d, J= 6.8 Hz, 4H), 8.24 (d, J= 6.8 Hz, 4H), 8.05 (d, J= 16.4 Hz, 2H), 7.84 (s, 4H), 7.62 (d, J= 16.4 Hz, 2H), 4.49 (t, J= 7.2 Hz, 4H), 1.88 (m, 4H), 1.25 (m, 36H), 0.81 (t, J= 7.2 Hz, 6H). ^13^C NMR (100 MHz, DMSO-*d*_6_) *δ*=152.93, 144.80, 140.19, 137.35, 129.22, 124.97, 124.47, 121.50, 118.30, 60.29, 31.72, 30.94, 29.42, 29.31, 29.19, 29.13, 28.80, 25.84, 22.51, 14.37. Anal. Calc for C_48_H_66_F_12_N_4_O_8_S_4_ (1183.30 g/mol): C, 48.72; H, 5.62; N, 4.73; S, 10.84%. Found C, 48.56; H, 5.74; N, 4.75; S, 11.04%

**2aTf_2_N**. (yellow solid 1.21 g, yield 72%). ^1^H NMR (400 MHz, DMSO-*d*_6_) *δ=*9.00 (d, J= 6 Hz, 2H), 8.57 (m, 4H), 8.01 (m, 8H), 7.79 (d, J= 16 Hz, 2H), 4.84 (t, J= 7.2 Hz, 4H), 1.86 (m, 4H), 1.36 (m, 12H), 0.86 (t, J= 6.8 Hz, 6H). ^13^C NMR (100 MHz, DMSO-*d*_6_) *δ*=151.99, 146.11, 145.14, 142.63, 137.24, 129.54, 126.48, 121.50, 118.97, 58.08, 30.96, 30.19, 25.51, 22.27, 14.18. Anal. Calc for C_36_H_42_F_12_N_4_O_8_S_4_ (1014.98 g/mol): C, 42.67; H, 4.14; N, 5.43; S, 12.82%. Found C, 42.90; H, 4.13; N, 5.53; S, 12.80%.

**2bTf_2_N**. (yellow solid 0.960 g, yield 64%). ^1^H NMR (400 MHz, DMSO-*d*_6_) *δ=*9.00 (d, J= 6 Hz, 2H), 8.57 (d, J= 7.2 Hz 4H), 8.00 (m, 8H), 7.79 (d, J= 16 Hz, 2H), 4.84 (t, J= 7.2 Hz, 4H), 1.85 (m, 4H), 1.31 (m, 24H), 0.82 (t, J= 7.2 Hz, 6H). ^13^C NMR (100 MHz, DMSO-*d*_6_) *δ*=152.00, 146.14, 145.16, 142.61, 137.24, 129.52, 126.50, 126.30, 121.50, 118.99, 58.06, 31.63, 30.20, 29.10, 28.94, 28.73, 25.77, 22.46, 14.34. Anal. Calc for C_42_H_54_F_12_N_4_O_8_S_4_ (1099.13 g/mol): C, 45.90; H, 4.95; N, 5.10; S, 11.67%. Found C, 45.20; H, 4.95; N, 5.11; S, 11.66%.

**2cTf_2_N**. (yellow solid 0.726 g, yield 86%). ^1^H NMR (400 MHz, DMSO-*d*_6_) *δ=*8.97 (d, J= 6 Hz, 2H), 8.53 (m, 4H), 7.97 (m, 8H), 7.60 (d, J= 16 Hz, 2H), 4.80 (t, J= 7.2 Hz, 4H), 1.81 (m, 4H), 1.27 (m, 36H), 0.79 (t, J= 6.8 Hz, 6H). ^13^C NMR (100 MHz, DMSO-*d*_6_) *δ*=151.99, 146.15, 145.16, 142.62, 137.24, 129.52, 126.46, 126.31, 118.96, 58.04, 31.70, 30.21, 29.43, 29.39, 29.29, 29.14, 28.73, 25.76, 22.50, 14.34. Anal. Calc for C_48_H_66_F_12_N_4_O_8_S_4_ (1183.30 g/mol): C, 48.72; H, 5.62; N, 4.73; S, 10.84%. Found C, 48.75; H, 5.69; N, 4.82; S, 10.65%

|  |  |
| --- | --- |


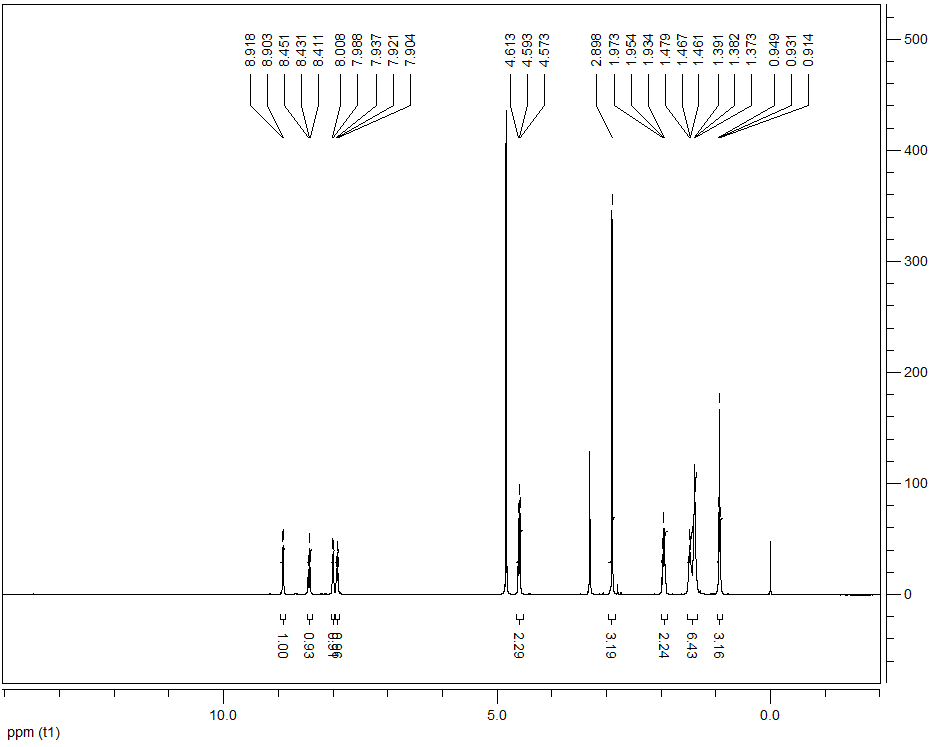

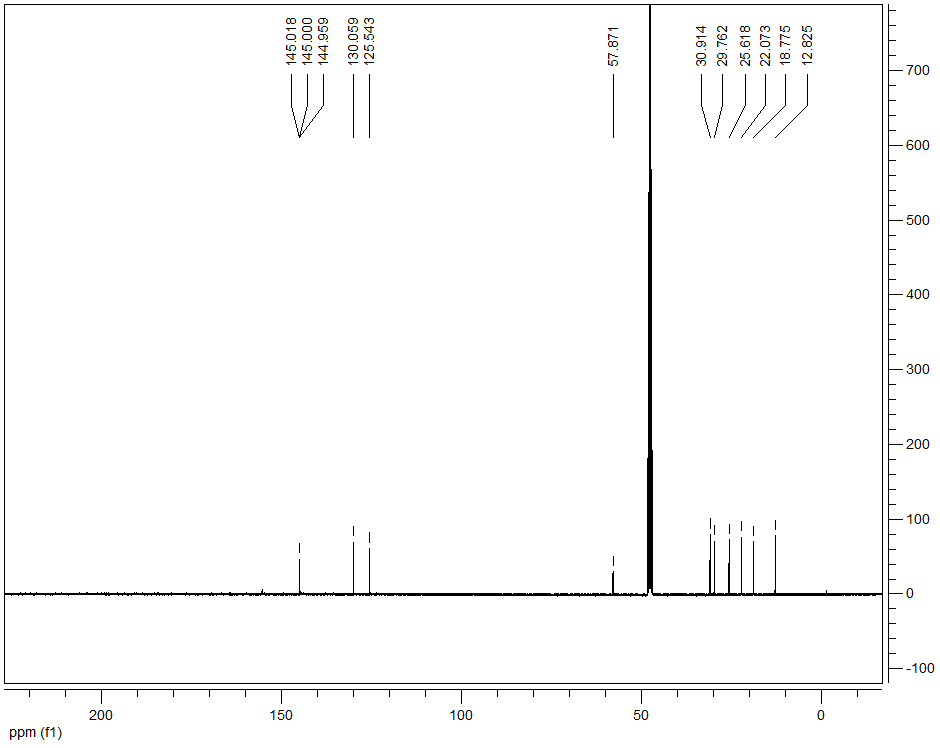


**Figure S1.** ^1^H and ^13^C NMR spectra of **2b** in CD_3_OD taken at room temperature.


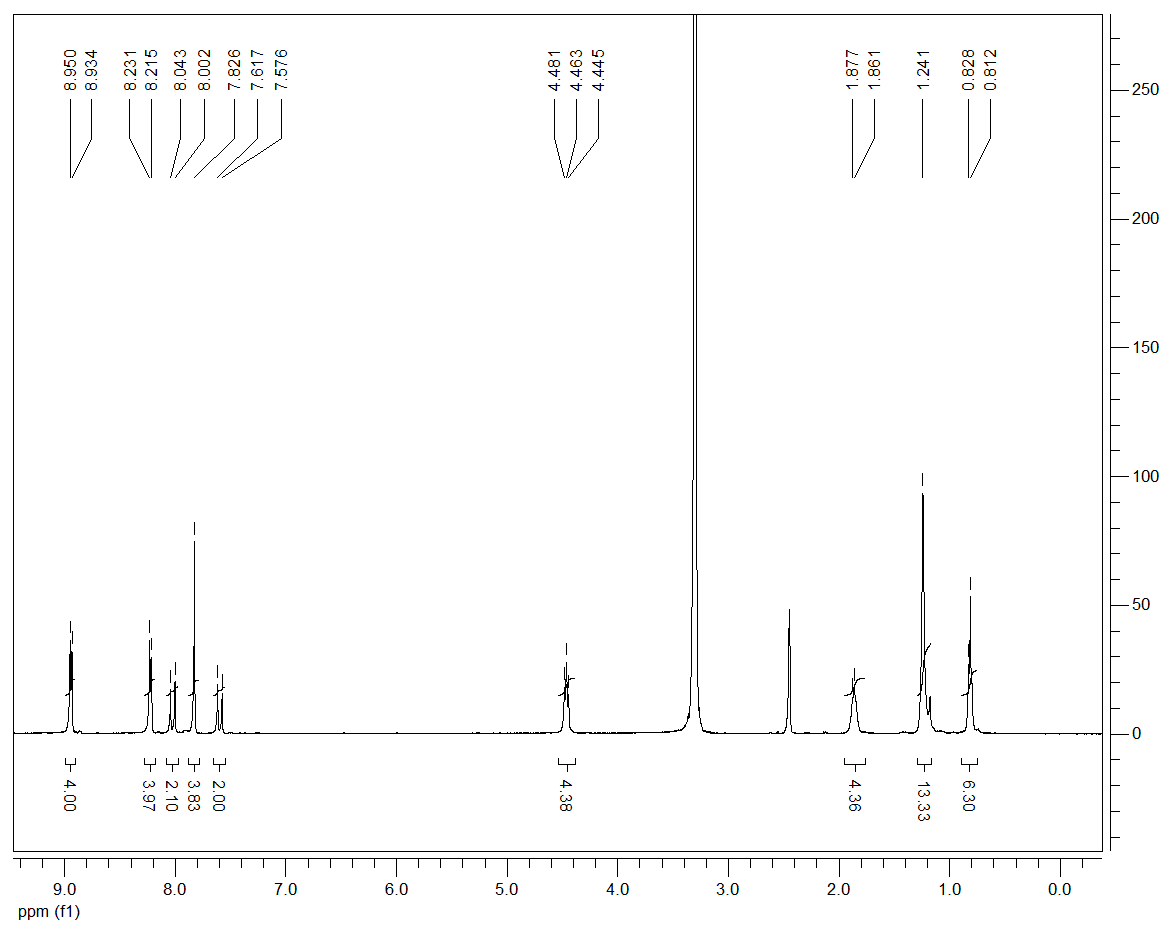


**Figure S2.** ^1^H NMR spectrum of **1aBr** in *d*_6_-DMSO taken at room temperature.


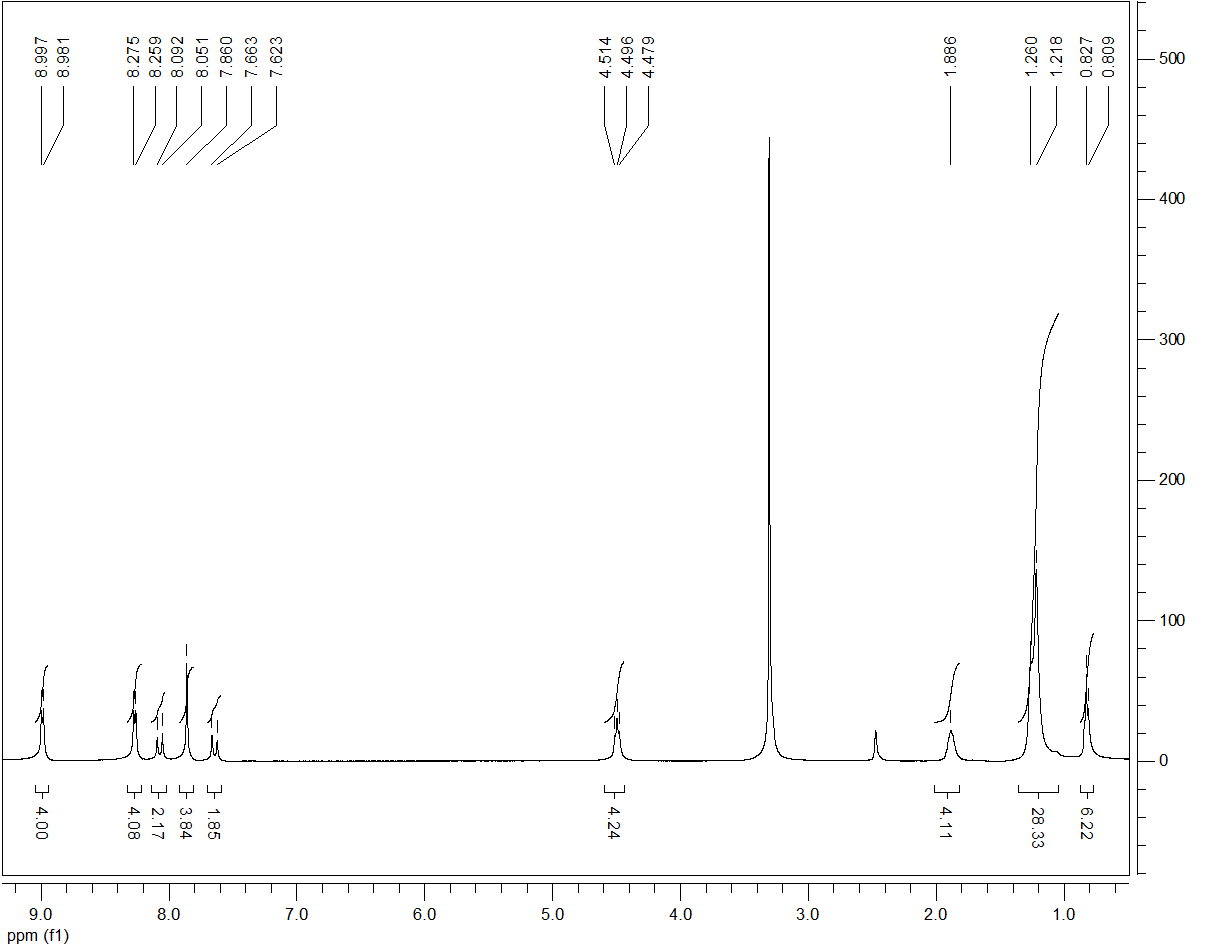


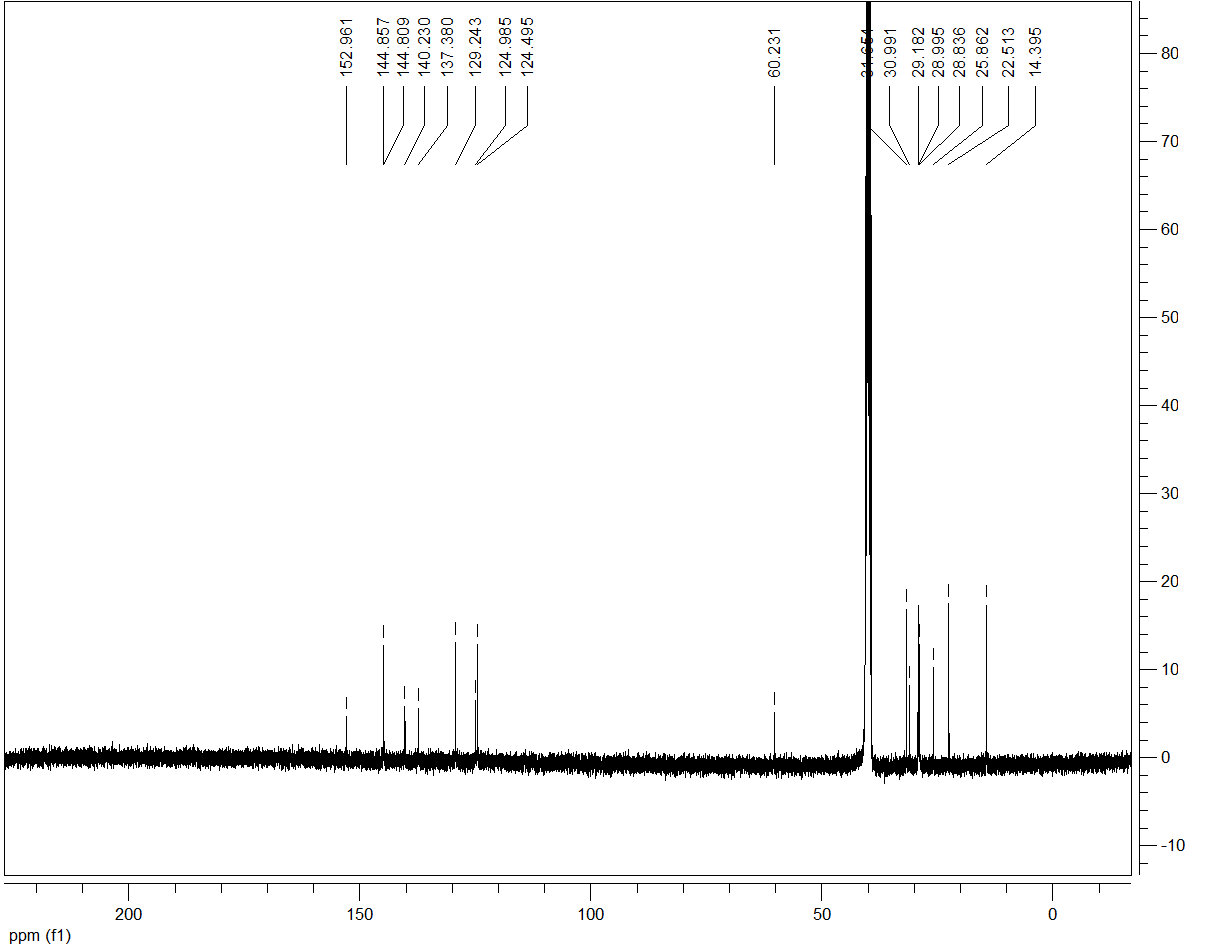


**Figure S3.** ^1^H and ^13^C NMR spectra of **1bBr** in *d*_6_-DMSO taken at room temperature.


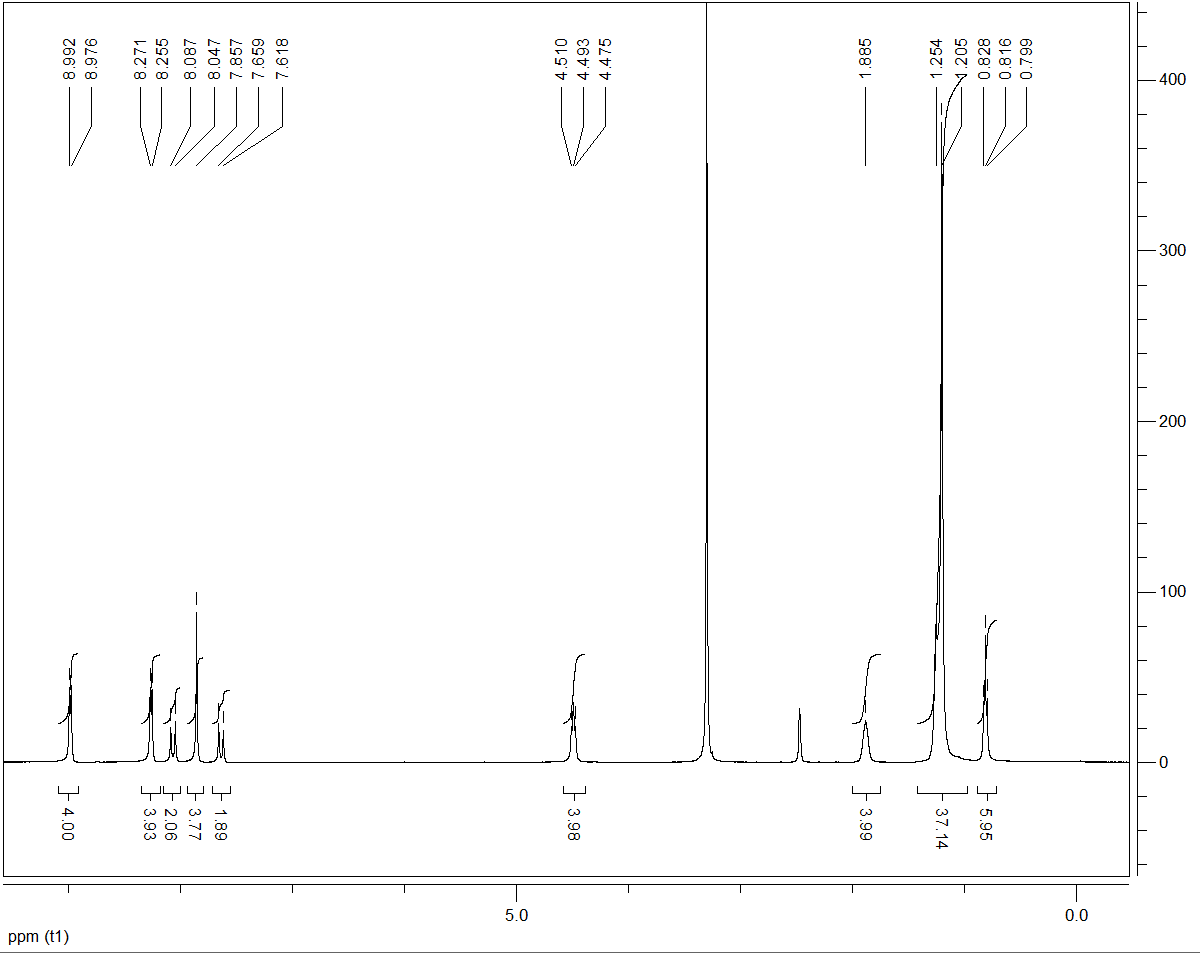


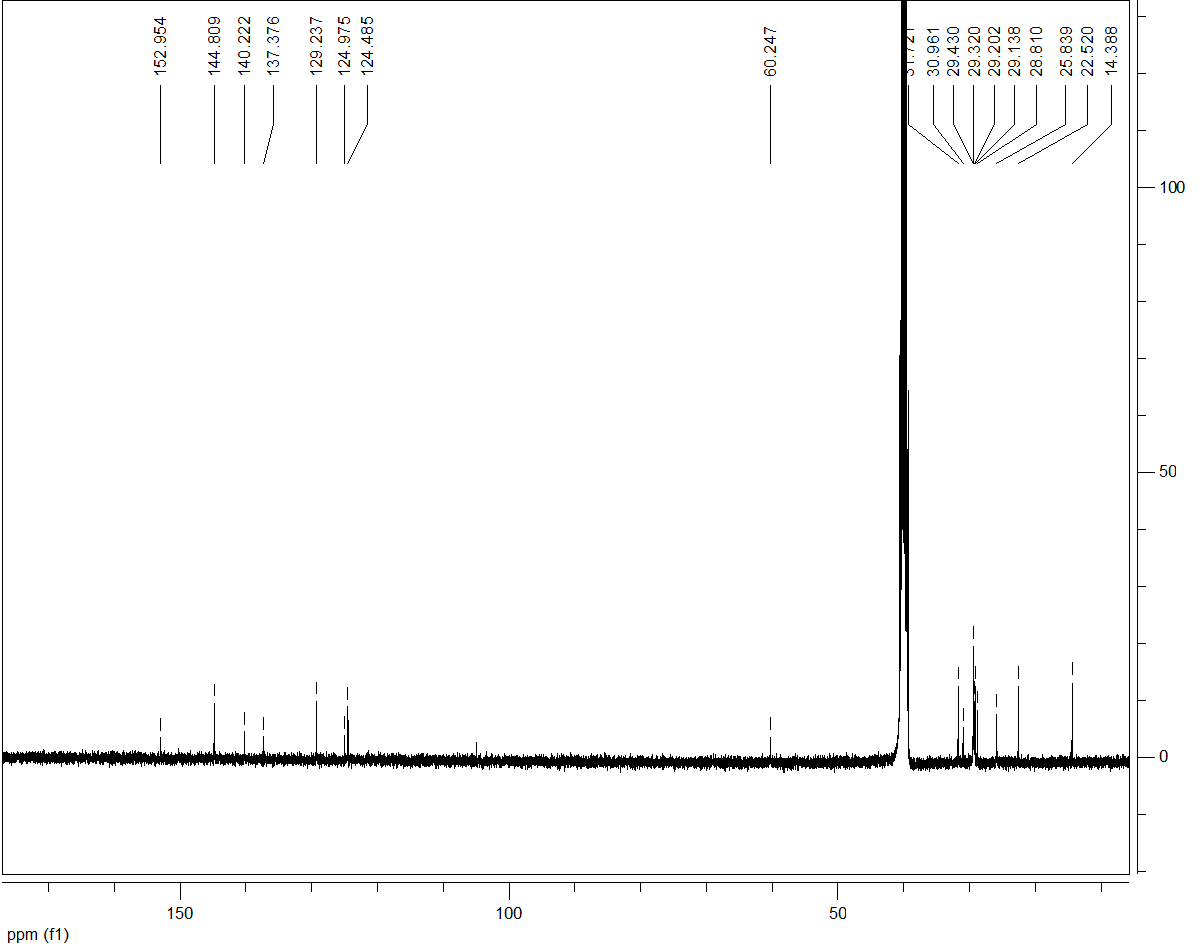


**Figure S4.** ^1^H and ^13^C NMR spectra of **1cBr** in *d*_6_-DMSO taken at room temperature.


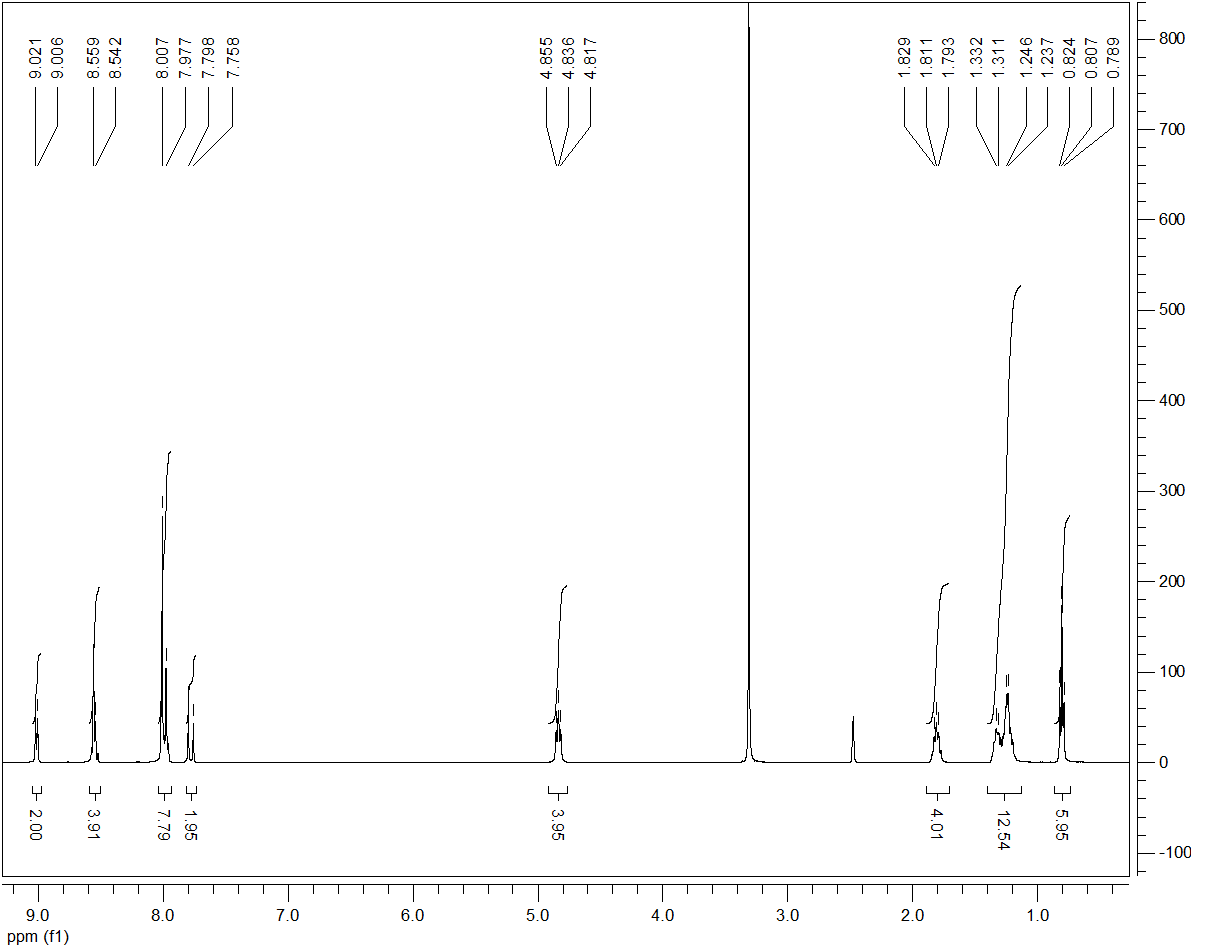


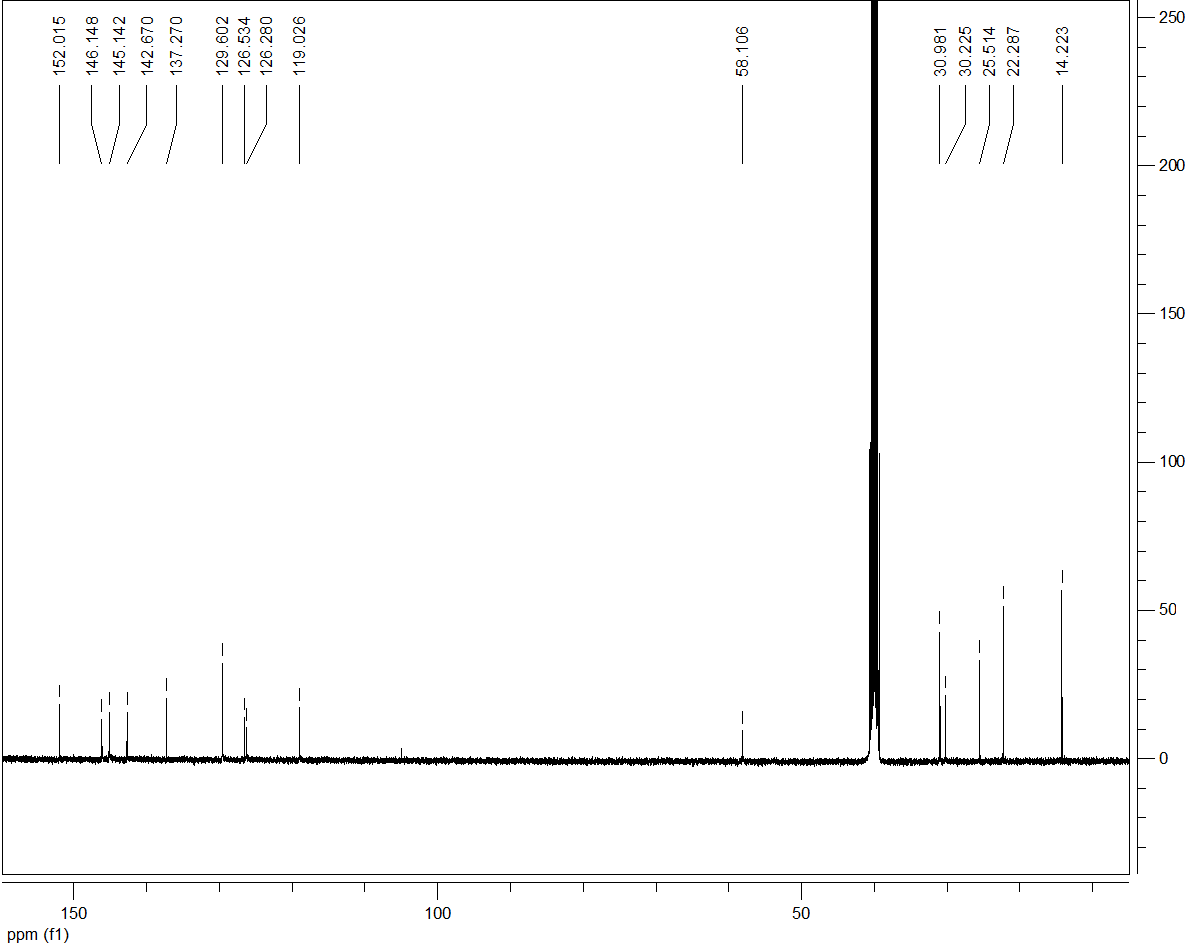


**Figure S5.** ^1^H and ^13^C NMR spectra of **2aBr** in *d*_6_-DMSO taken at room temperature.


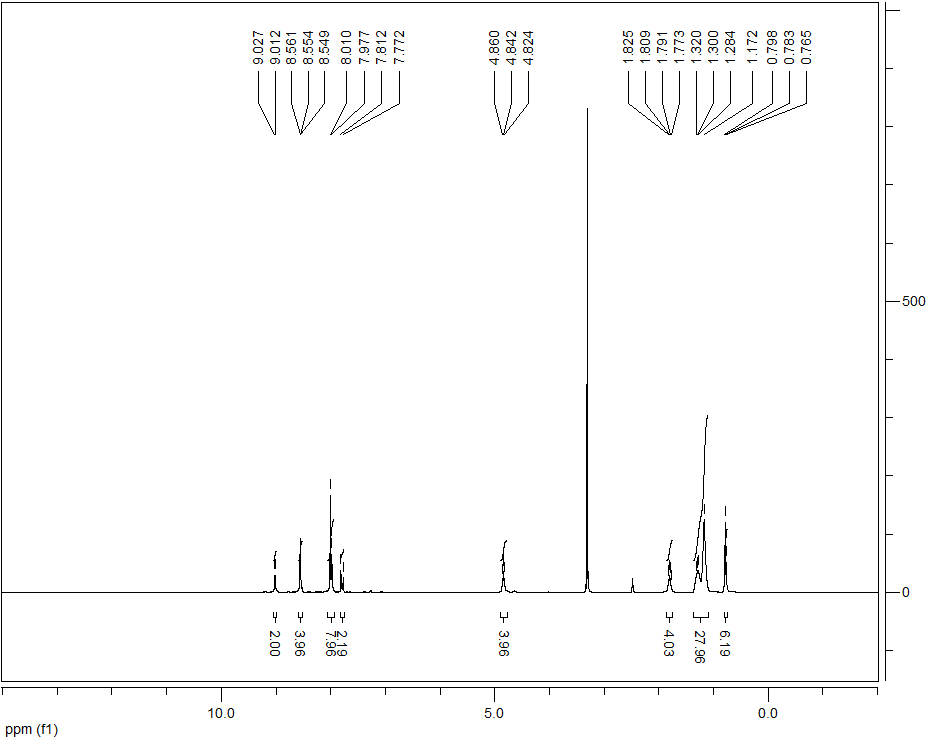


**
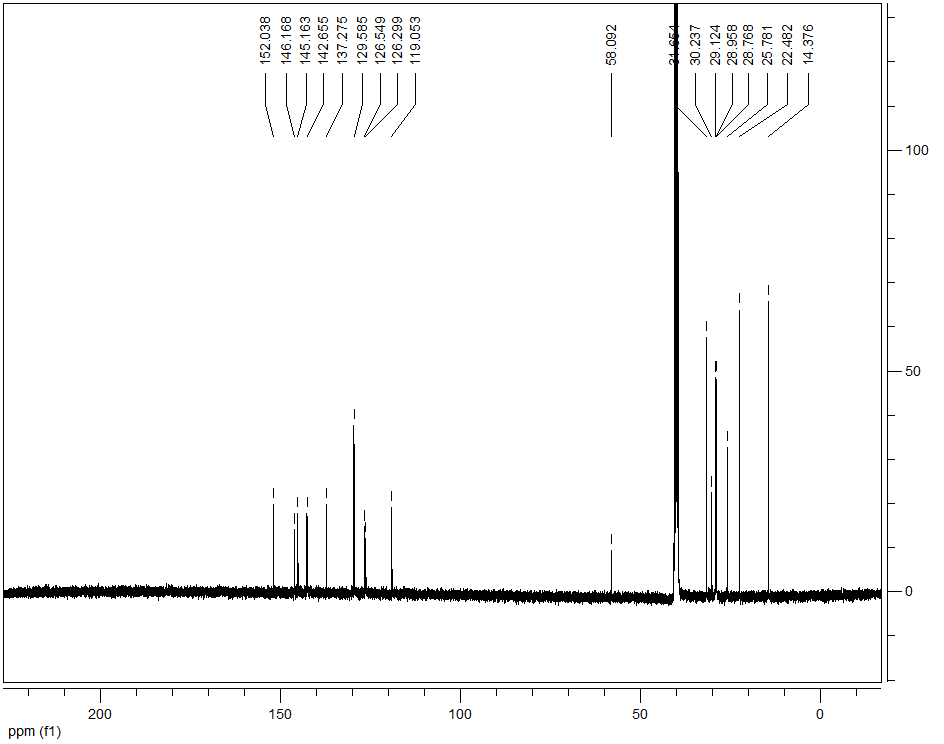
**

**Figure S6.** ^1^H and ^13^C NMR spectra of **2bBr** in *d*_6_-DMSO taken at room temperature.


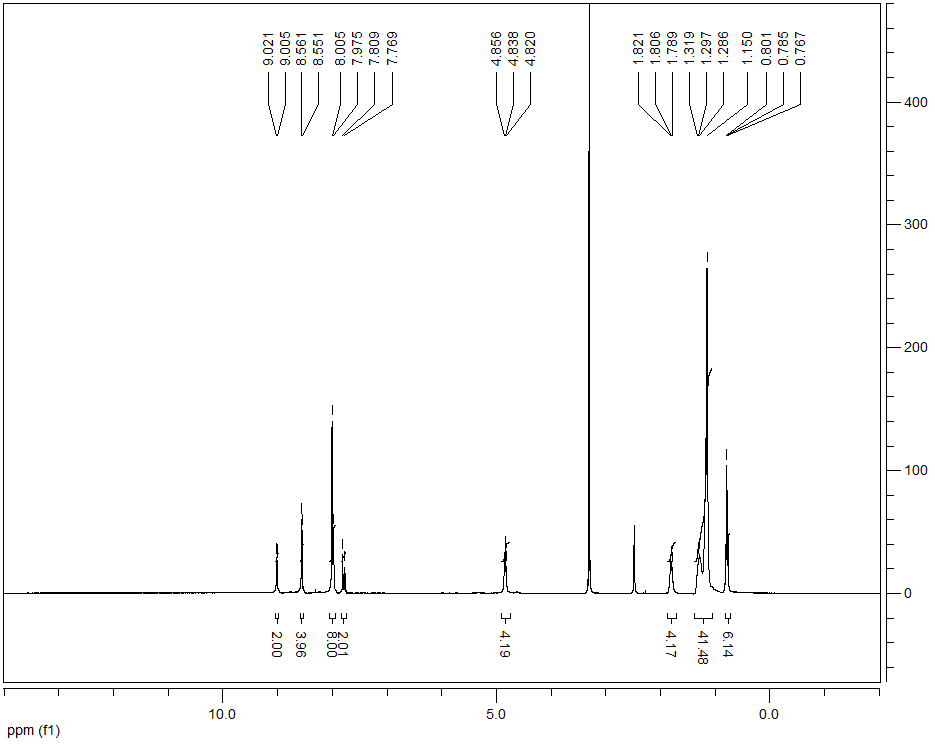

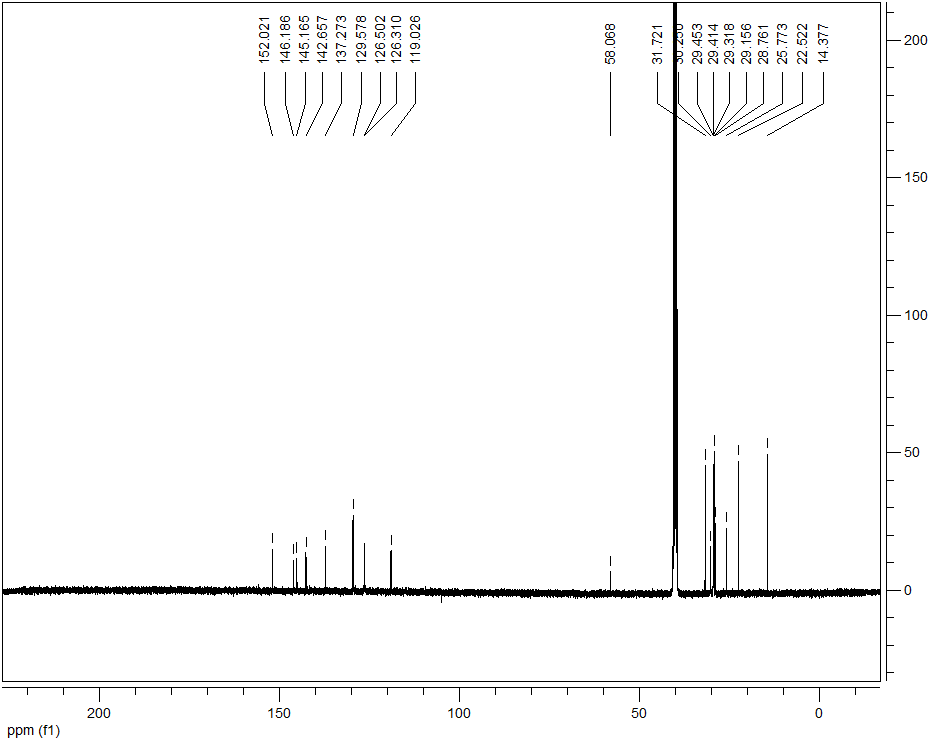


**Figure S7.** ^1^H and ^13^C NMR spectra of **2cBr** in *d*_6_-DMSO taken at room temperature.


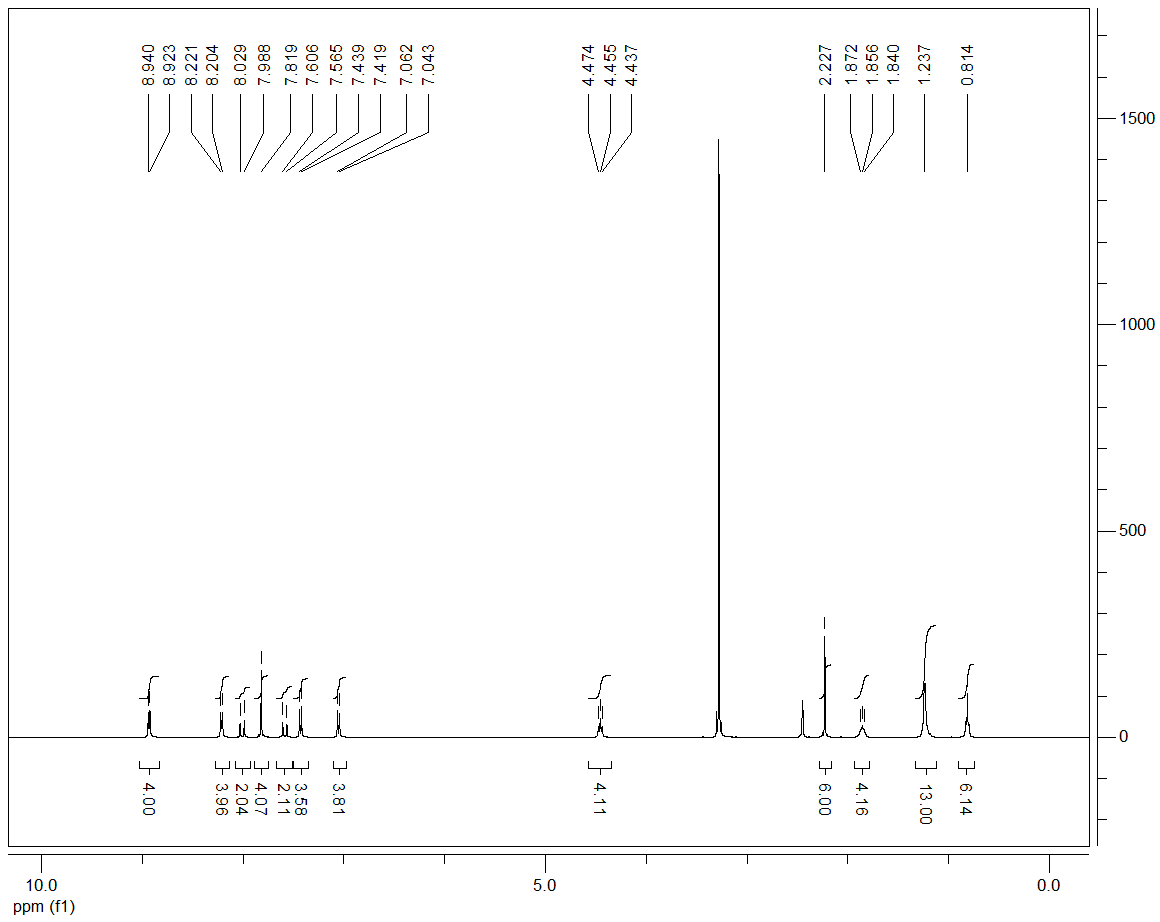

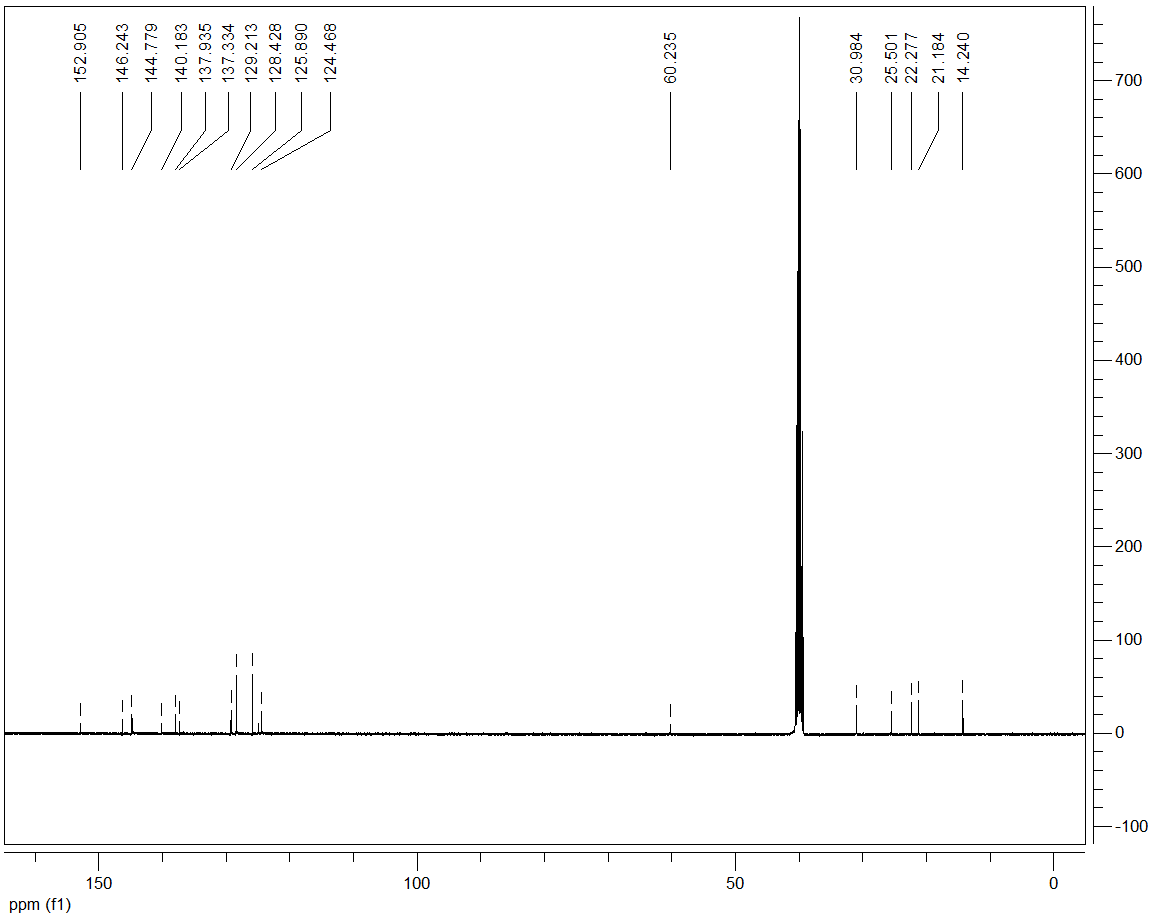


**Figure S8.** ^1^H and ^13^C NMR spectra of **1aOTs** in *d*_6_-DMSO taken at room temperature.


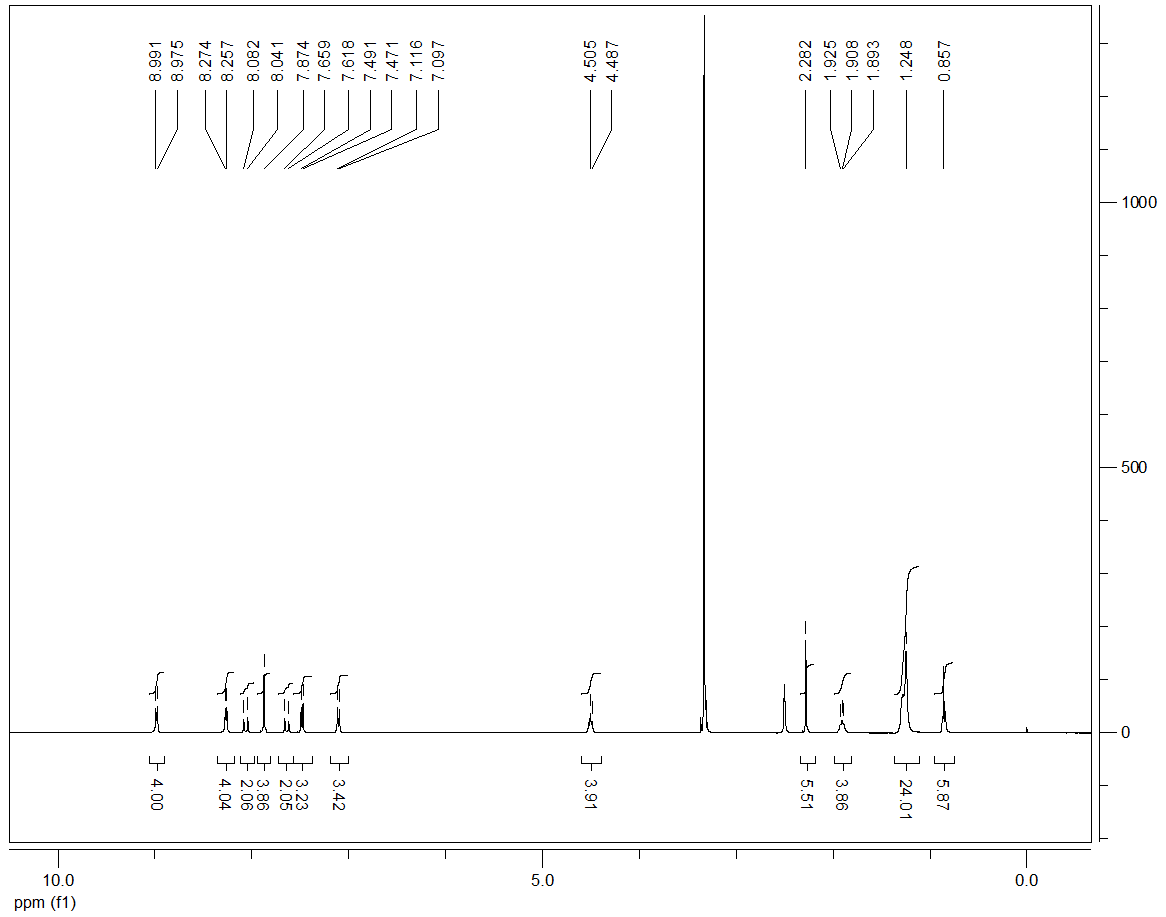


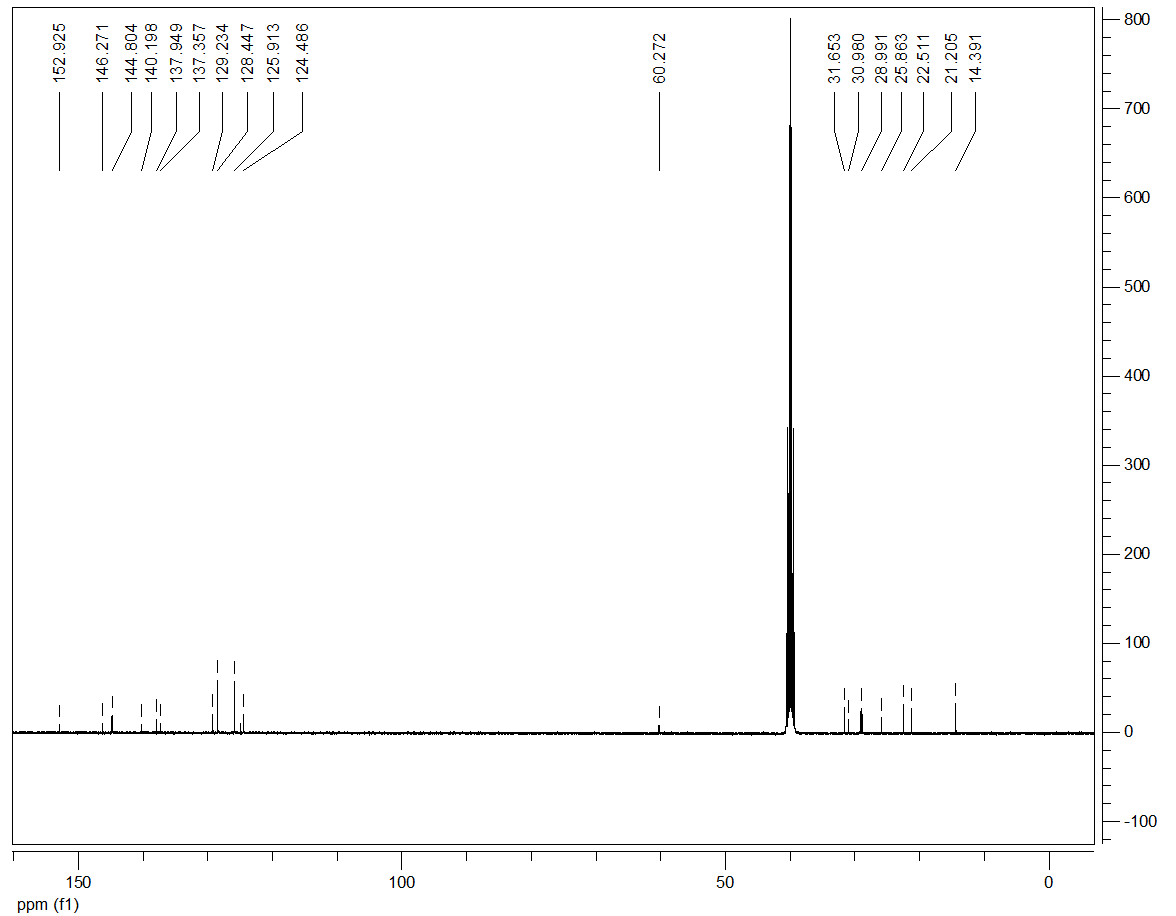


**Figure S9.** ^1^H and ^13^C NMR spectra of **1bOTs** in *d*_6_-DMSO taken at room temperature.


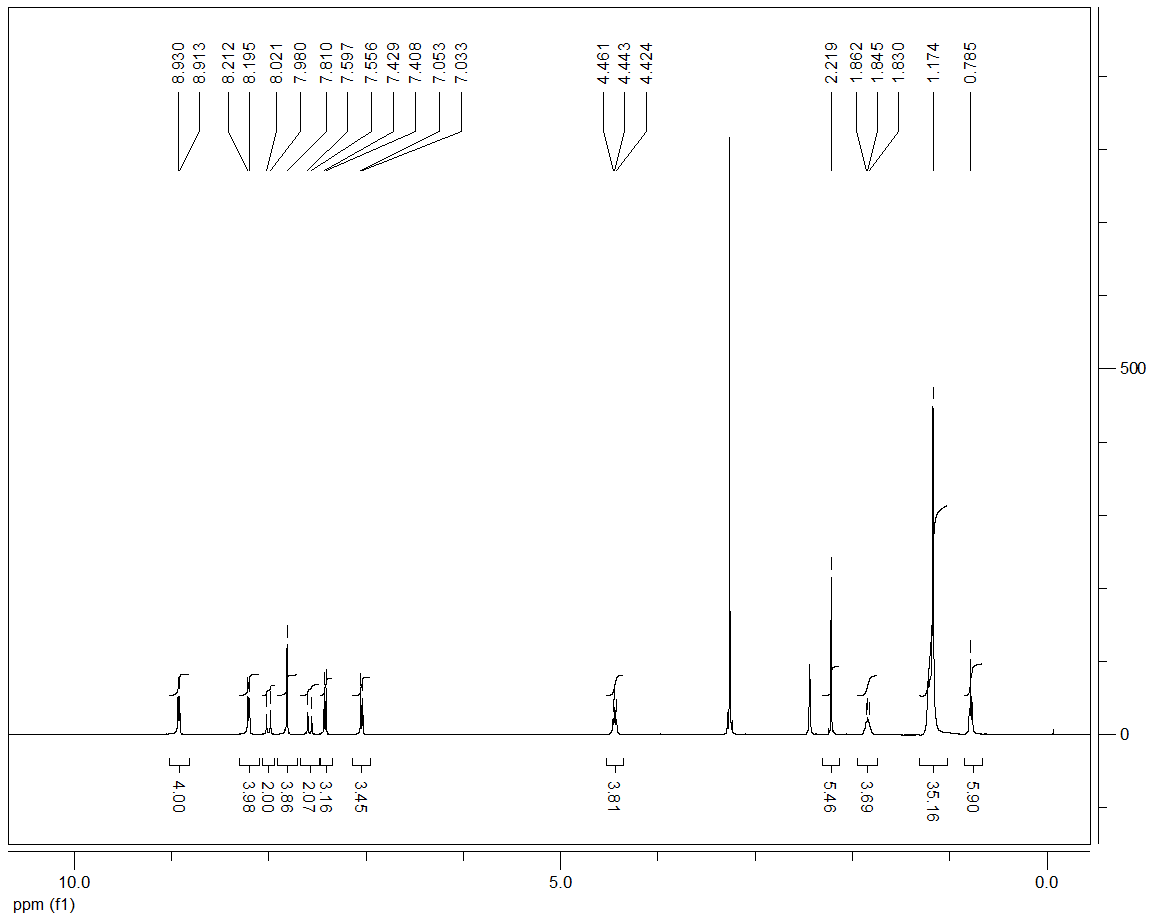


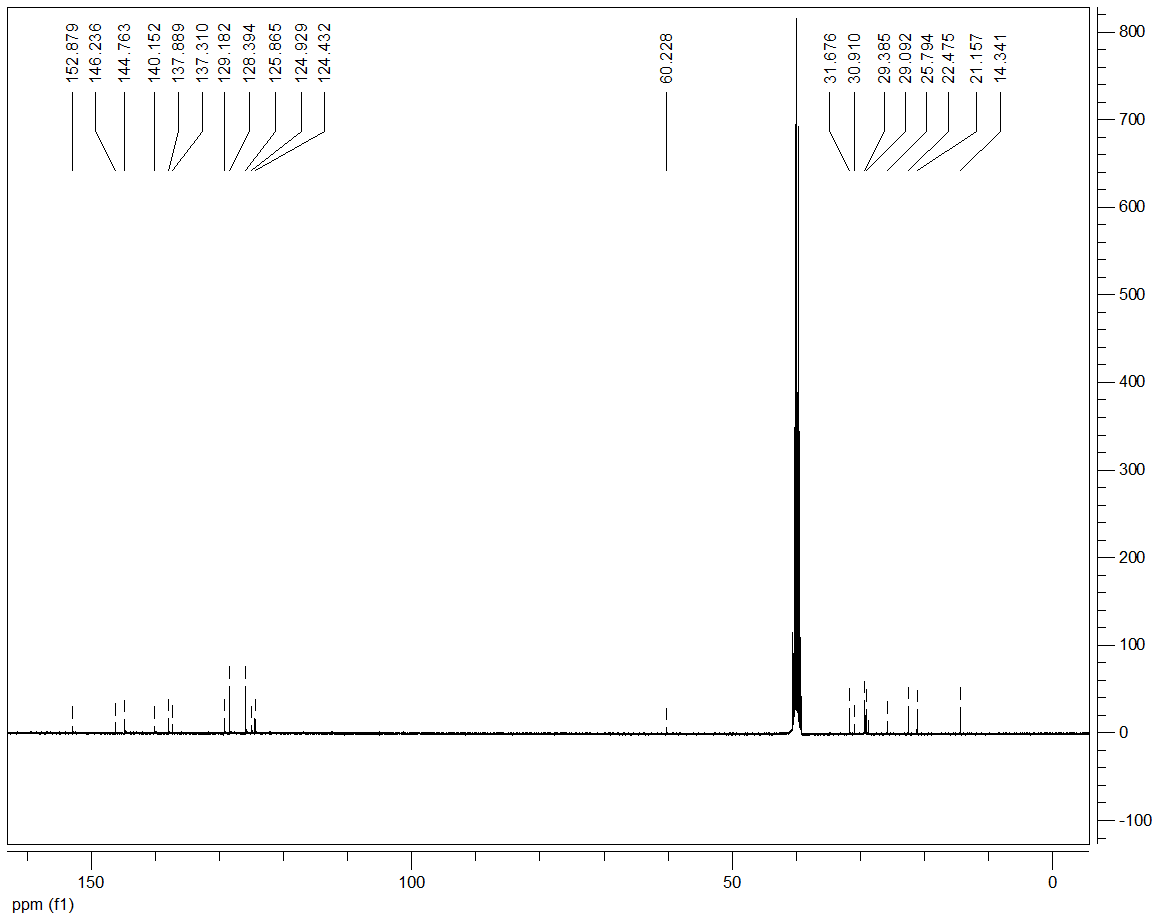


**Figure S10.** ^1^H and ^13^C NMR spectra of **1cOTs** in *d*_6_-DMSO taken at room temperature.


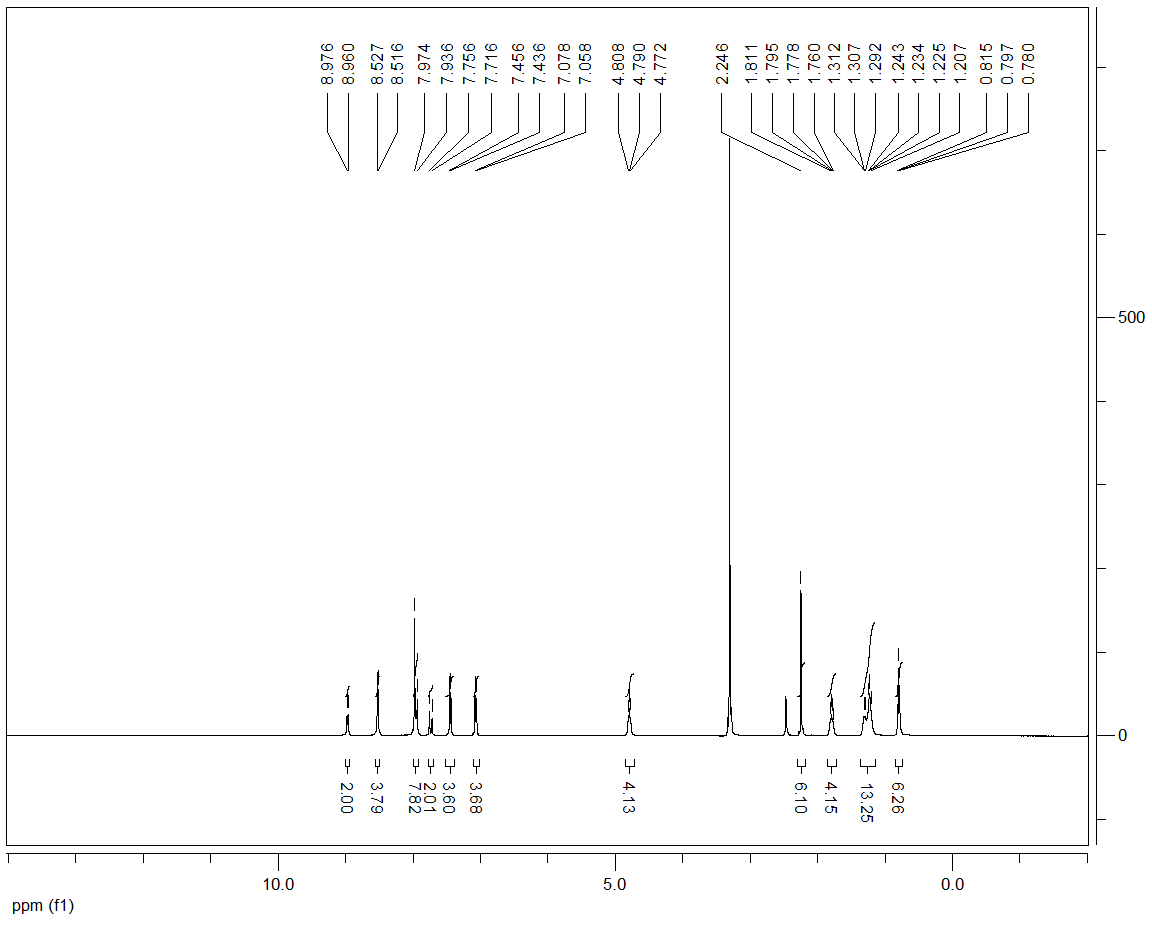


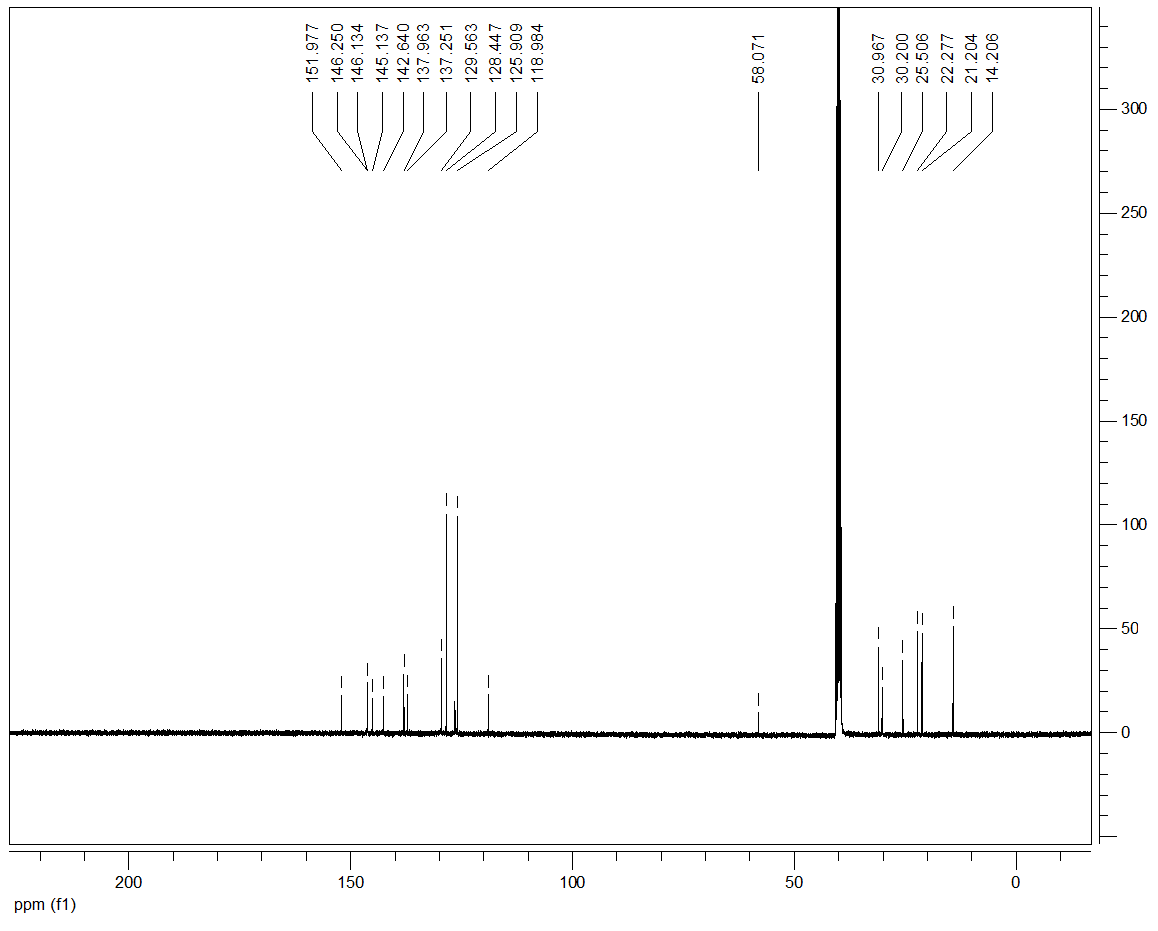


**Figure S11.** ^1^H and ^13^C NMR spectra of **2aOTs** in *d*_6_-DMSO taken at room temperature.


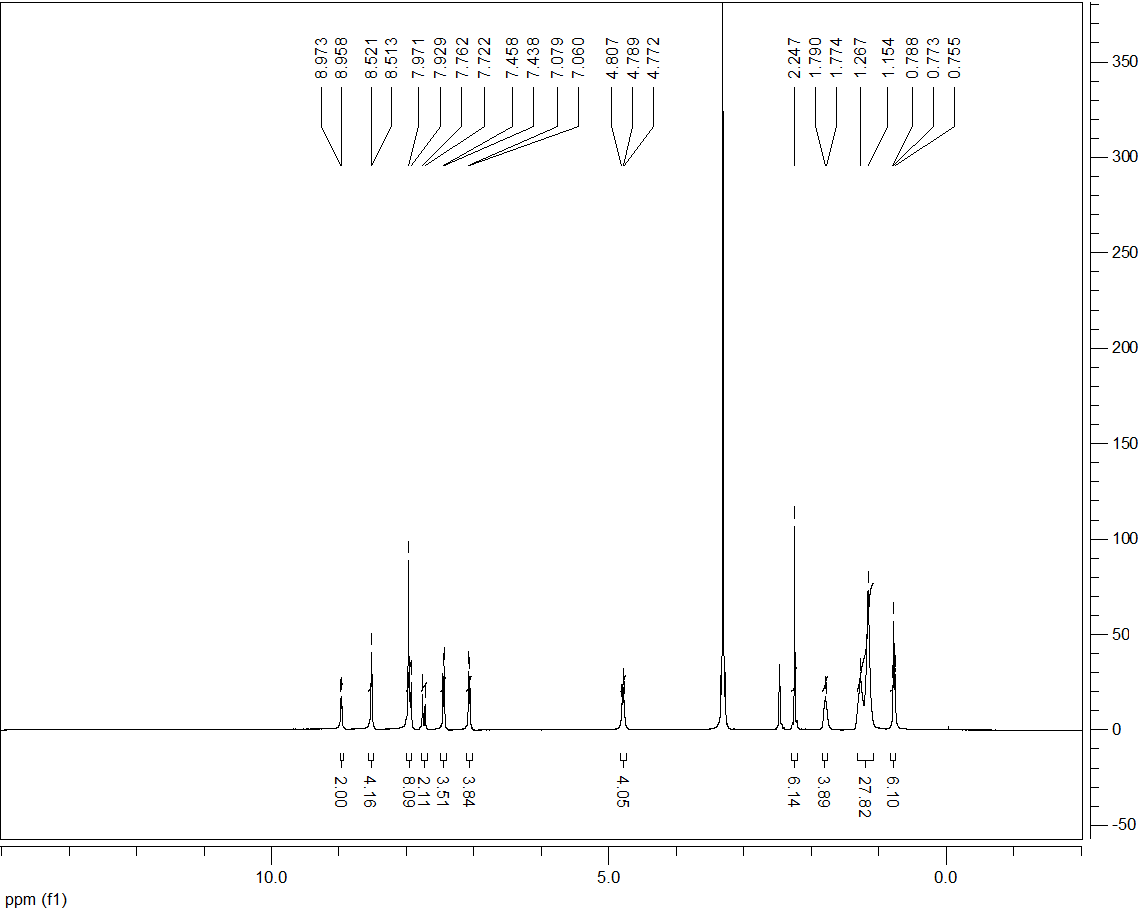


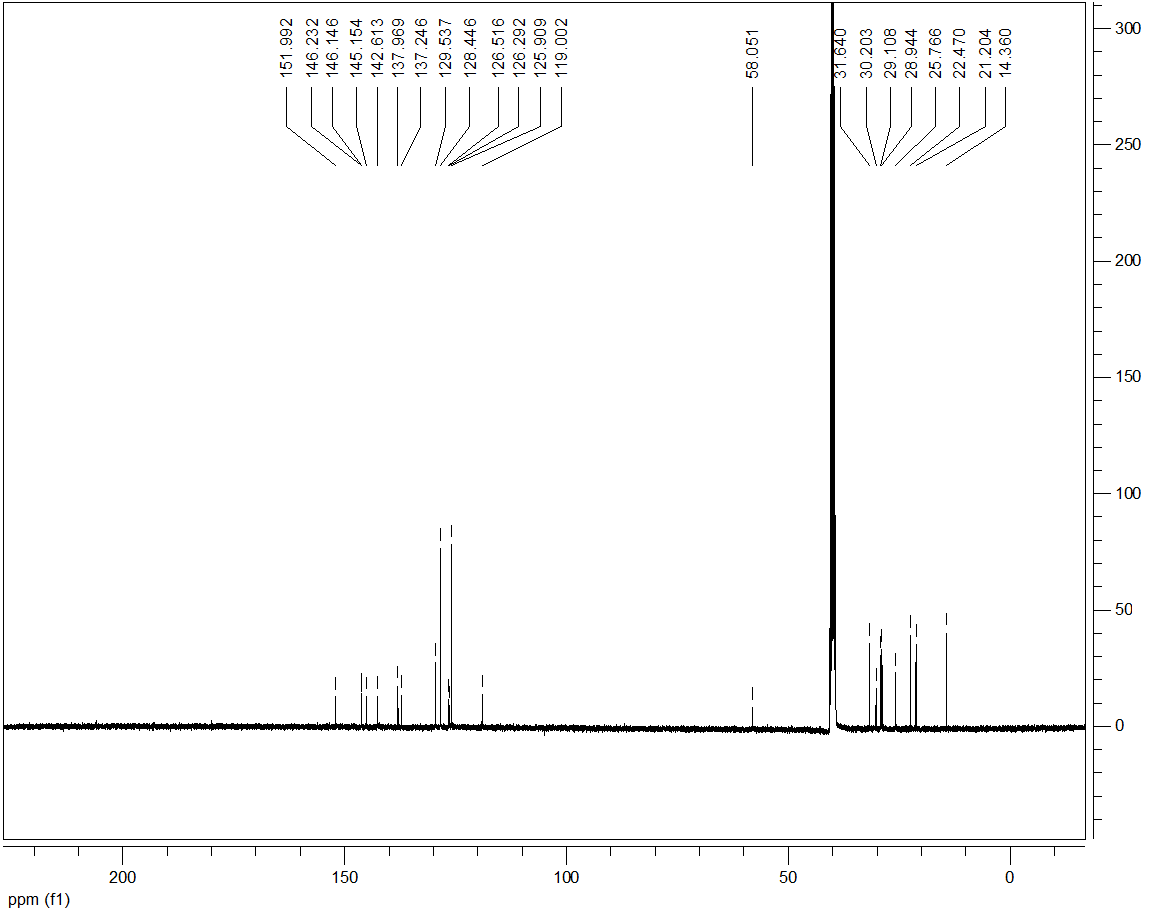


**Figure S12.** ^1^H and ^13^C NMR spectra of **2bOTs** in *d*_6_-DMSO taken at room temperature.


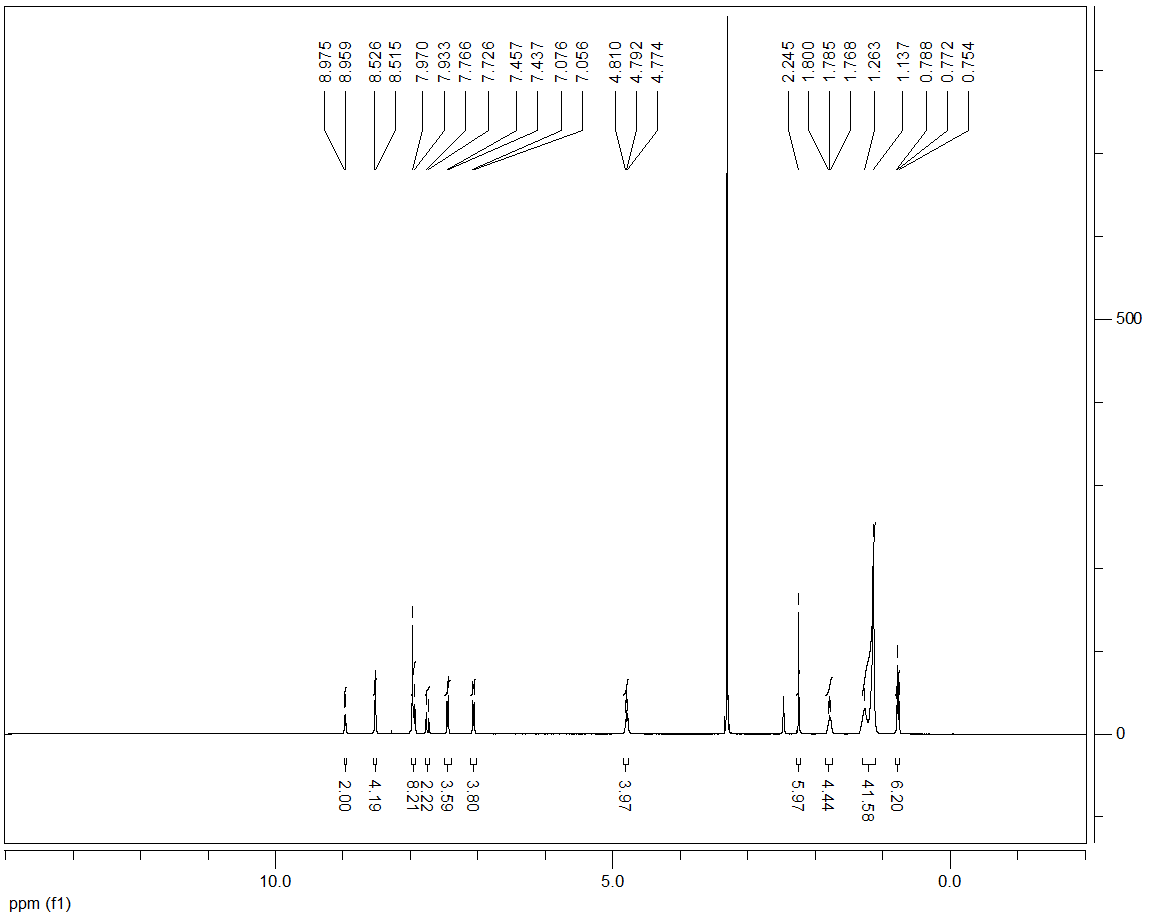


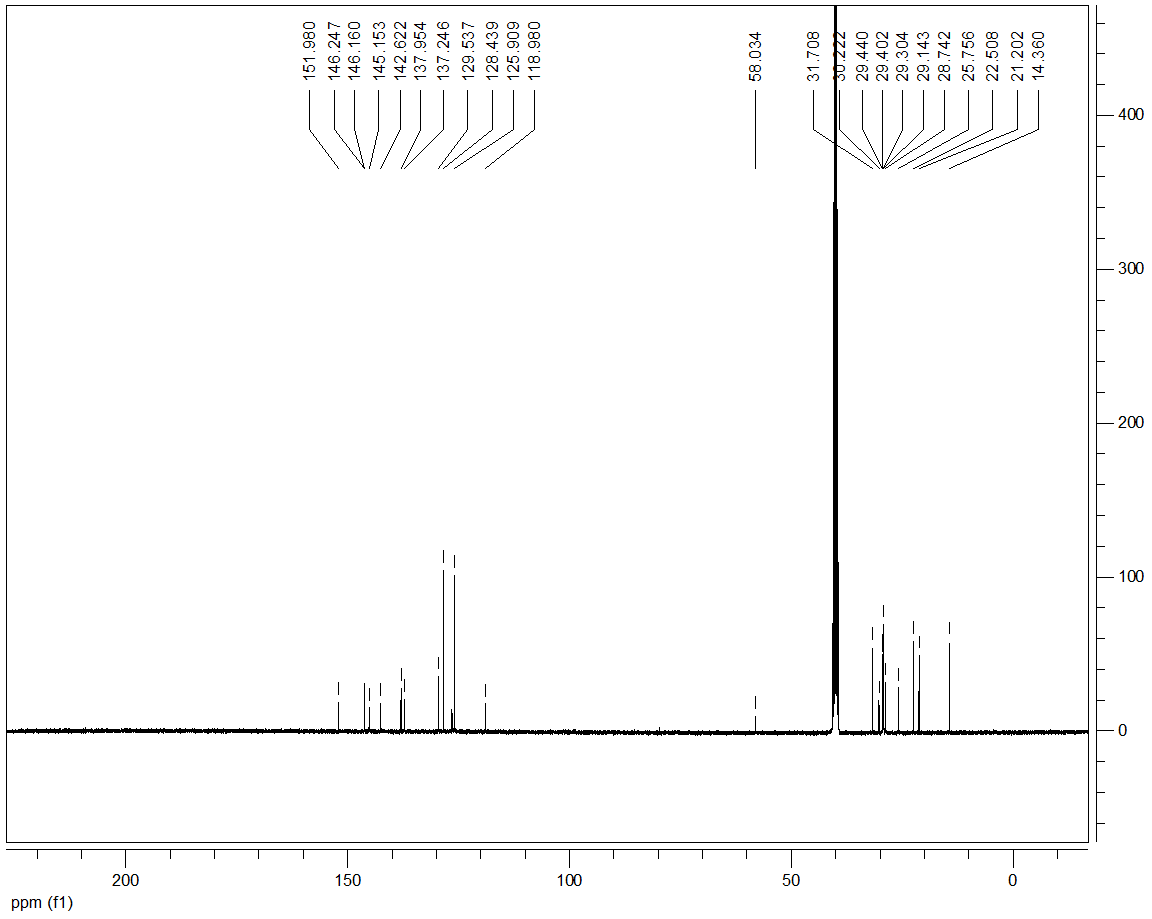


**Figure S13.** ^1^H and ^13^C NMR spectra of **2cOTs** in *d*_6_-DMSO taken at room temperature.


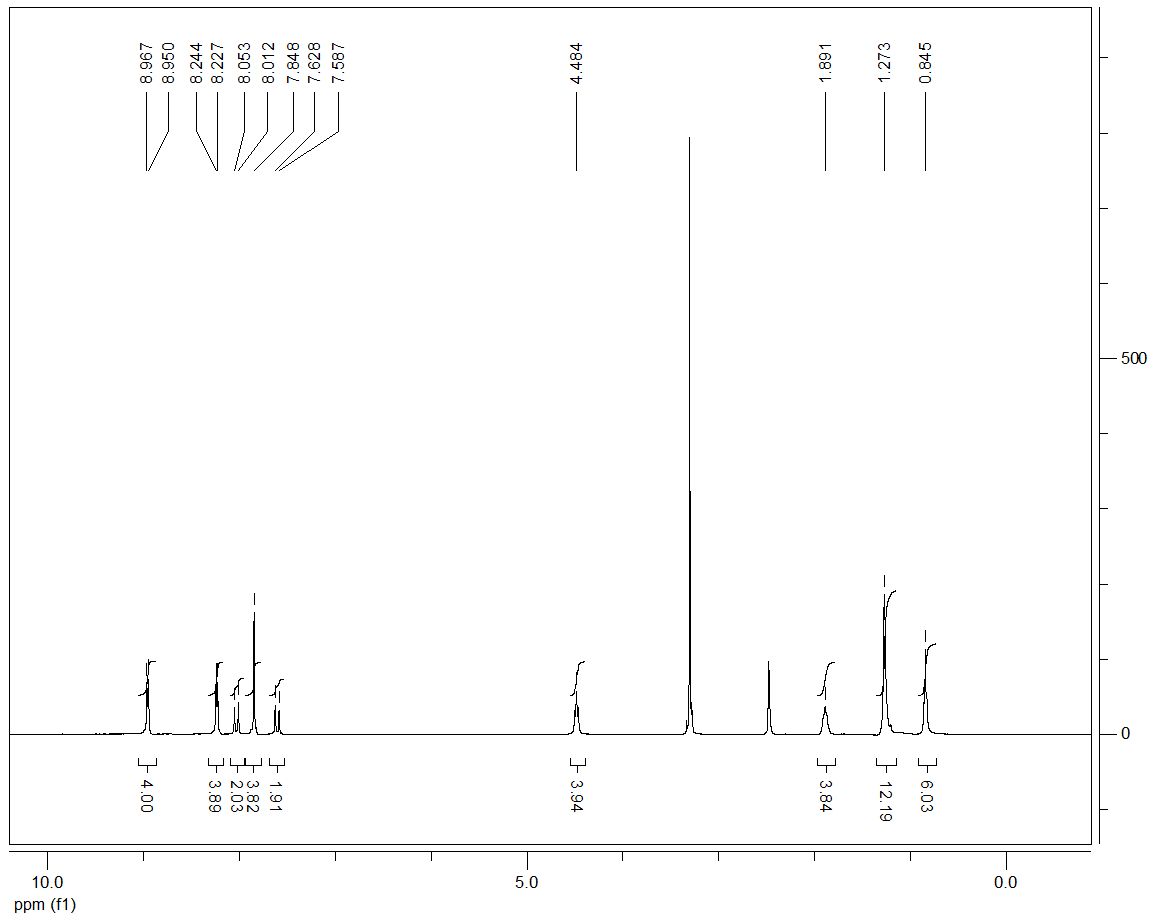


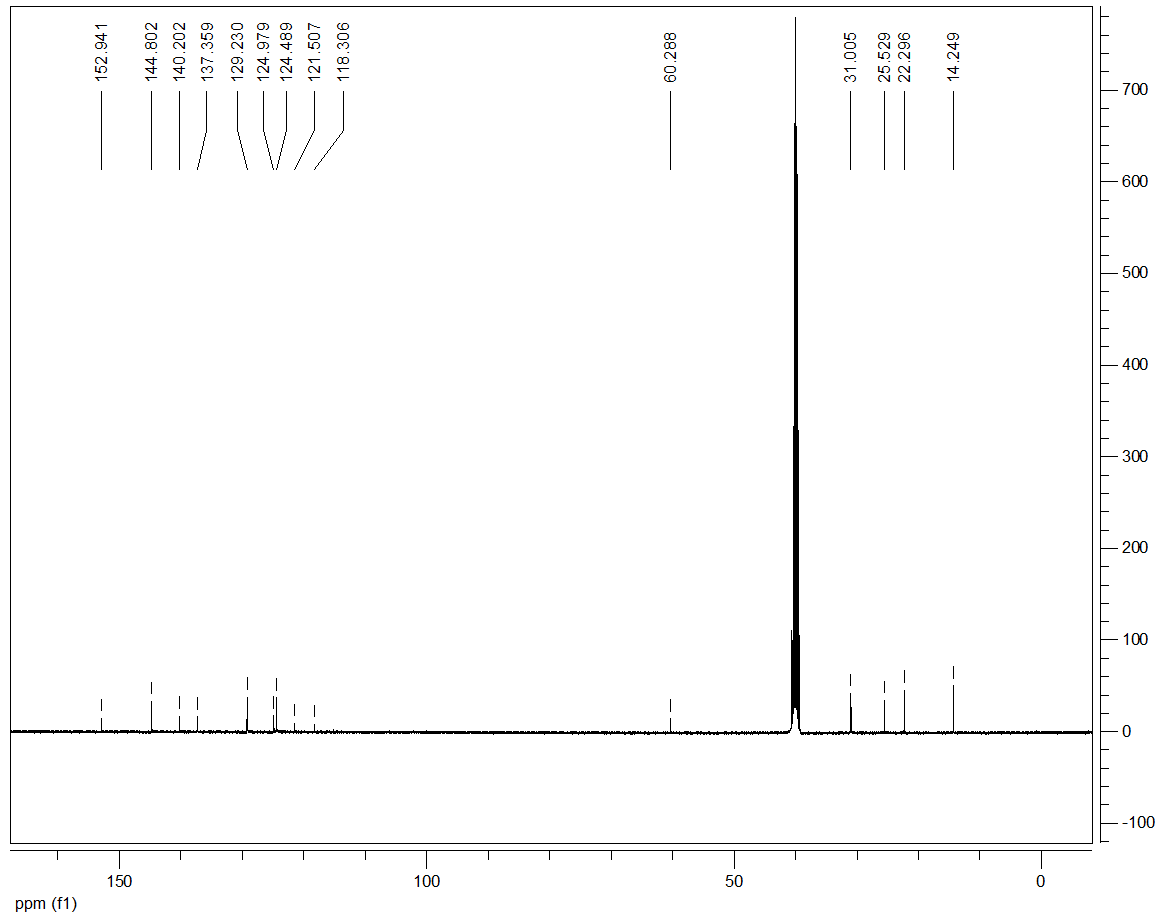


**Figure S14.** ^1^H and ^13^C NMR spectra of **1aTf_2_N** in *d*_6_-DMSO taken at room temperature.


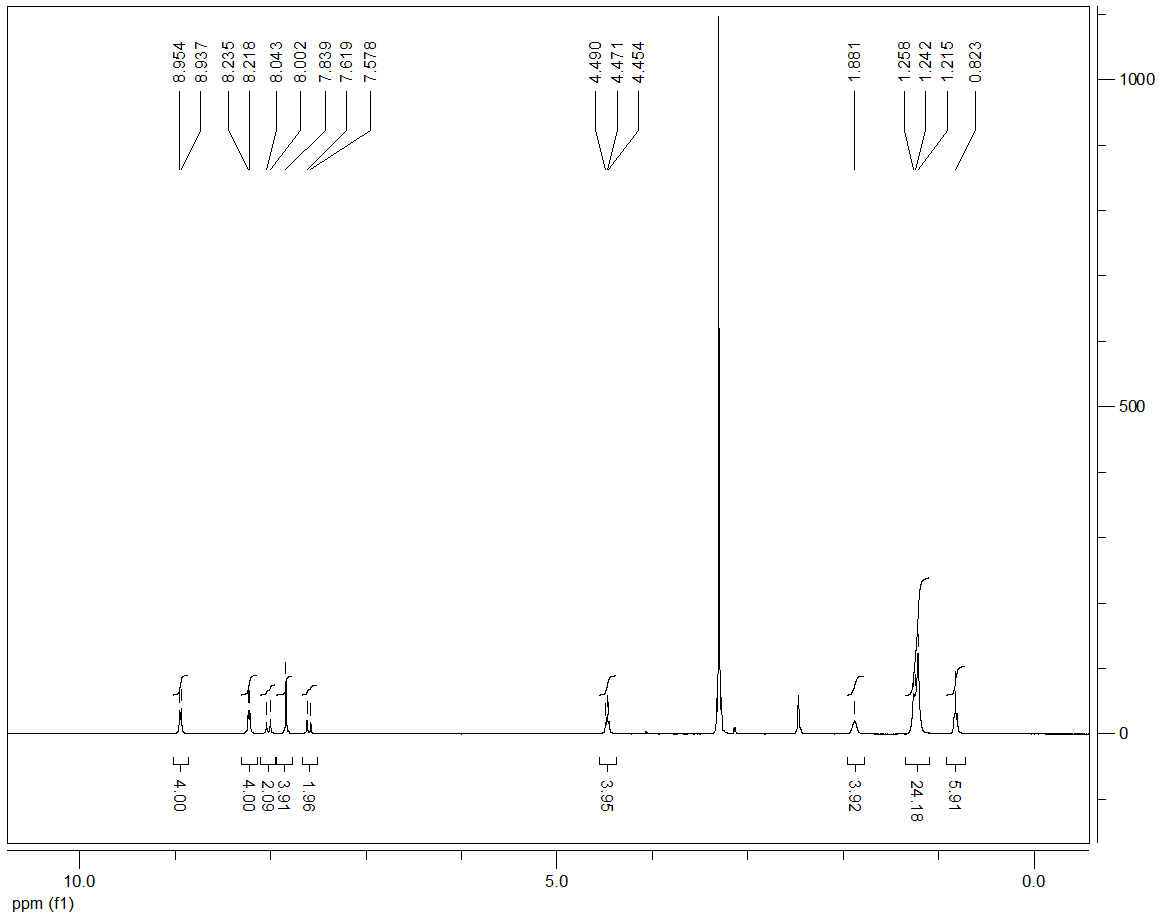


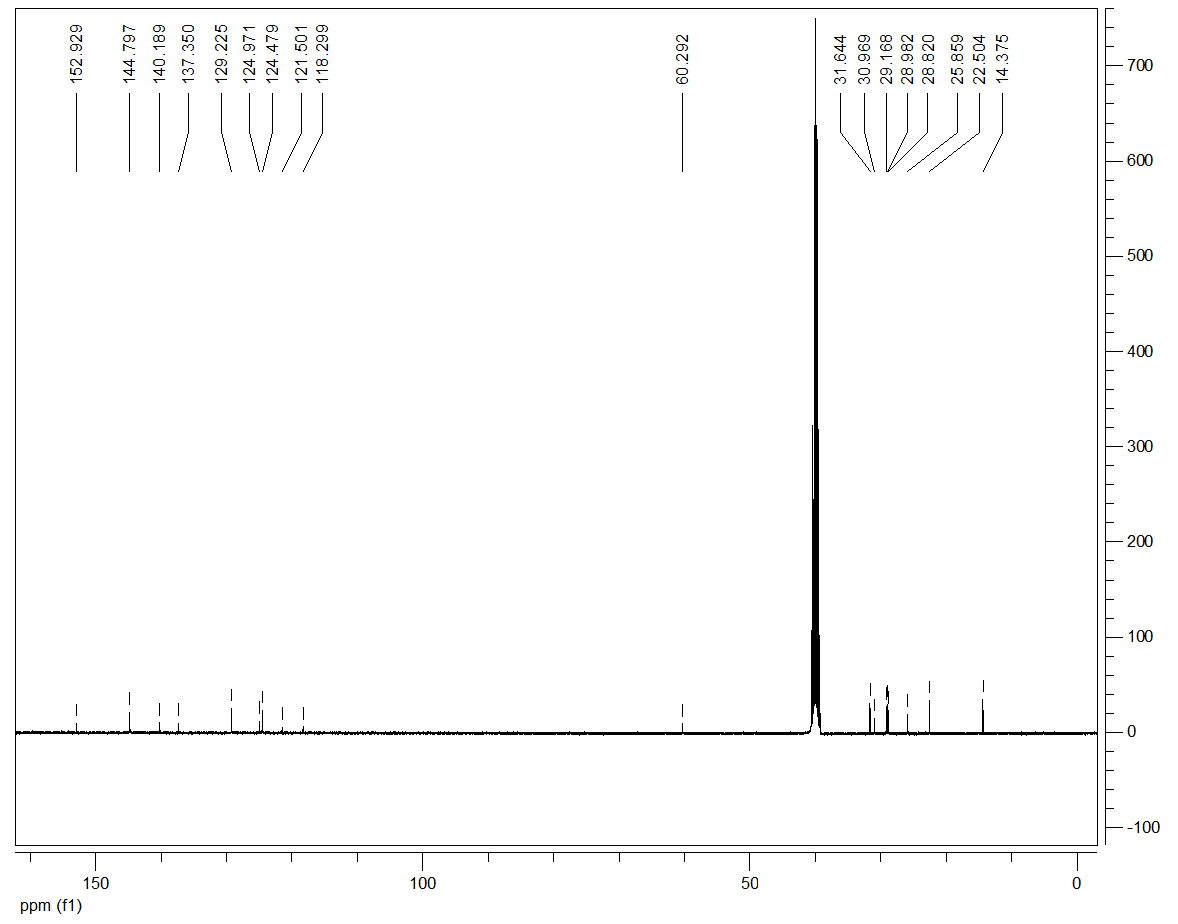


**Figure S15.** ^1^H and ^13^C NMR spectra of **1bTf_2_N** in *d*_6_-DMSO taken at room temperature.


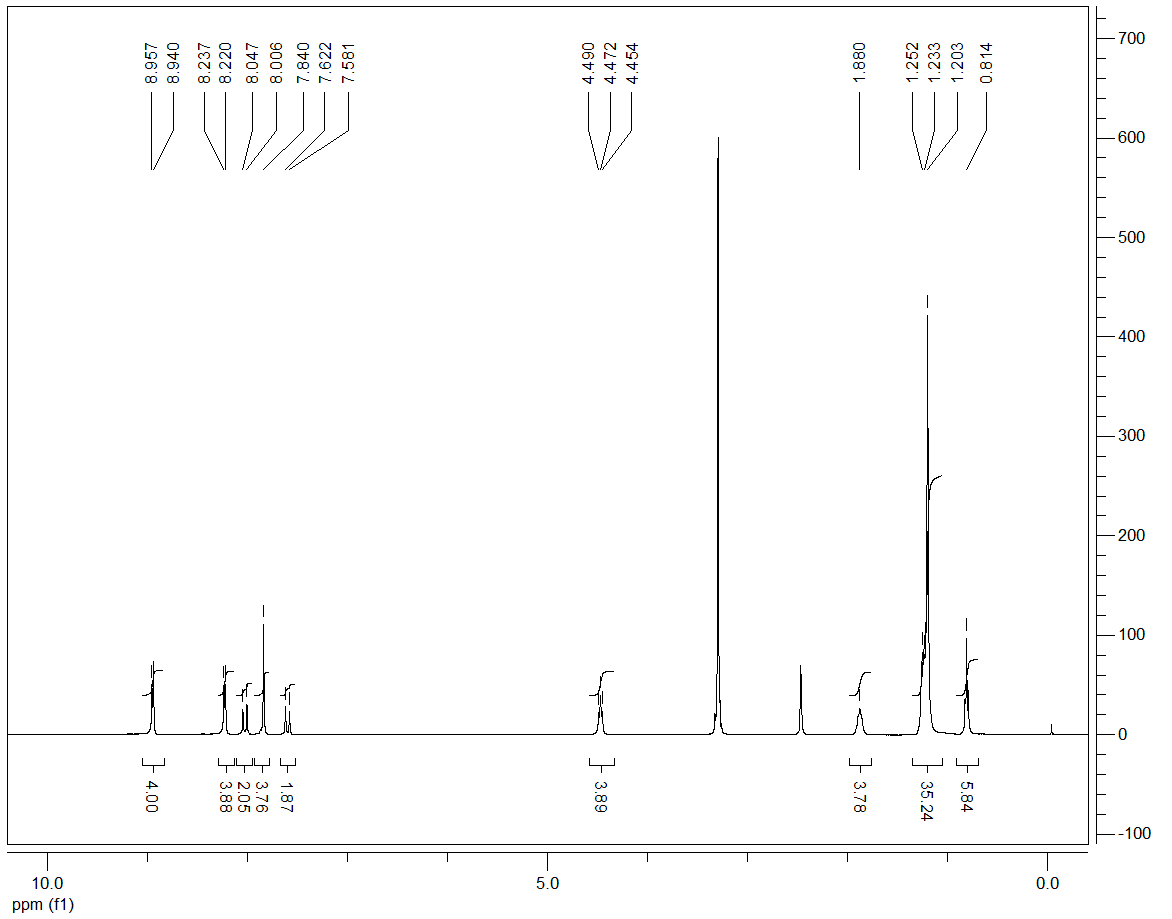


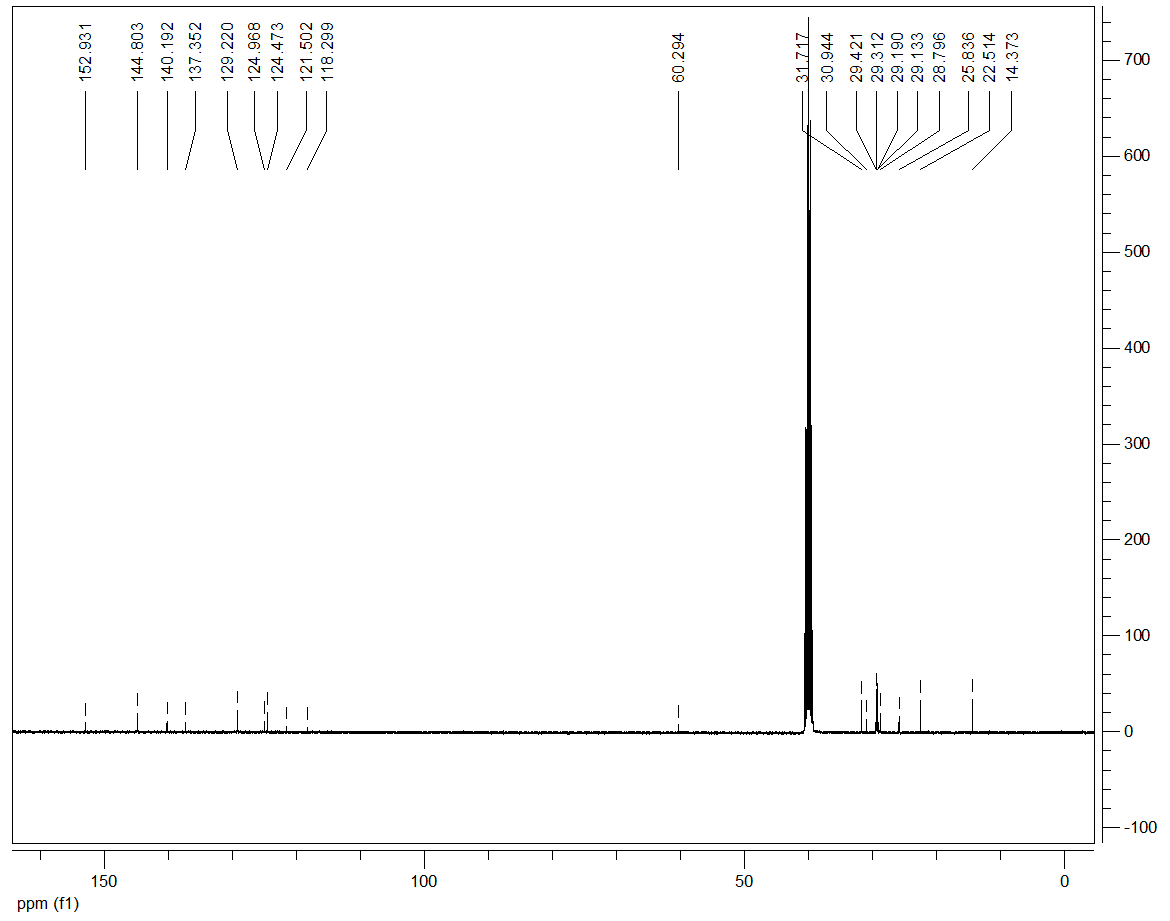


**Figure S16.** ^1^H and ^13^C NMR spectra of **1cTf_2_N** in *d*_6_-DMSO taken at room temperature.


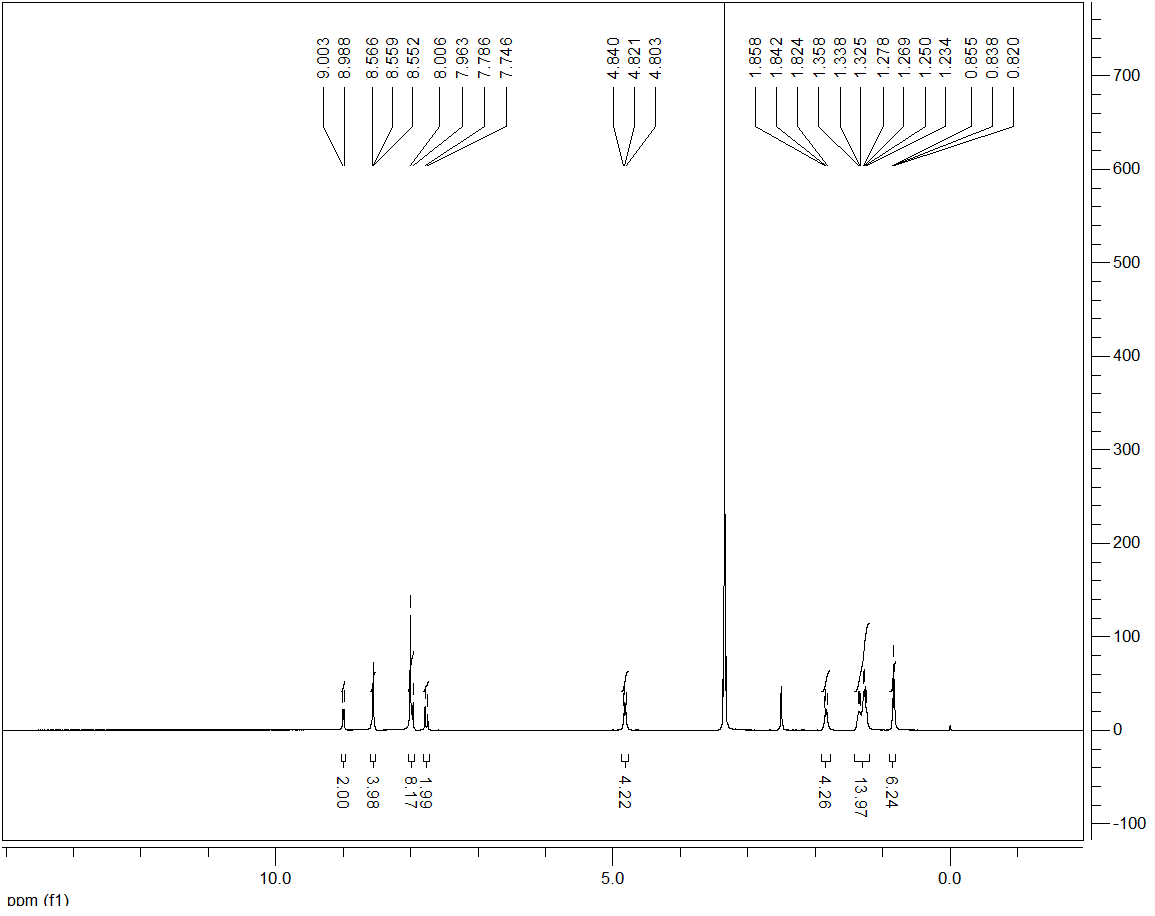

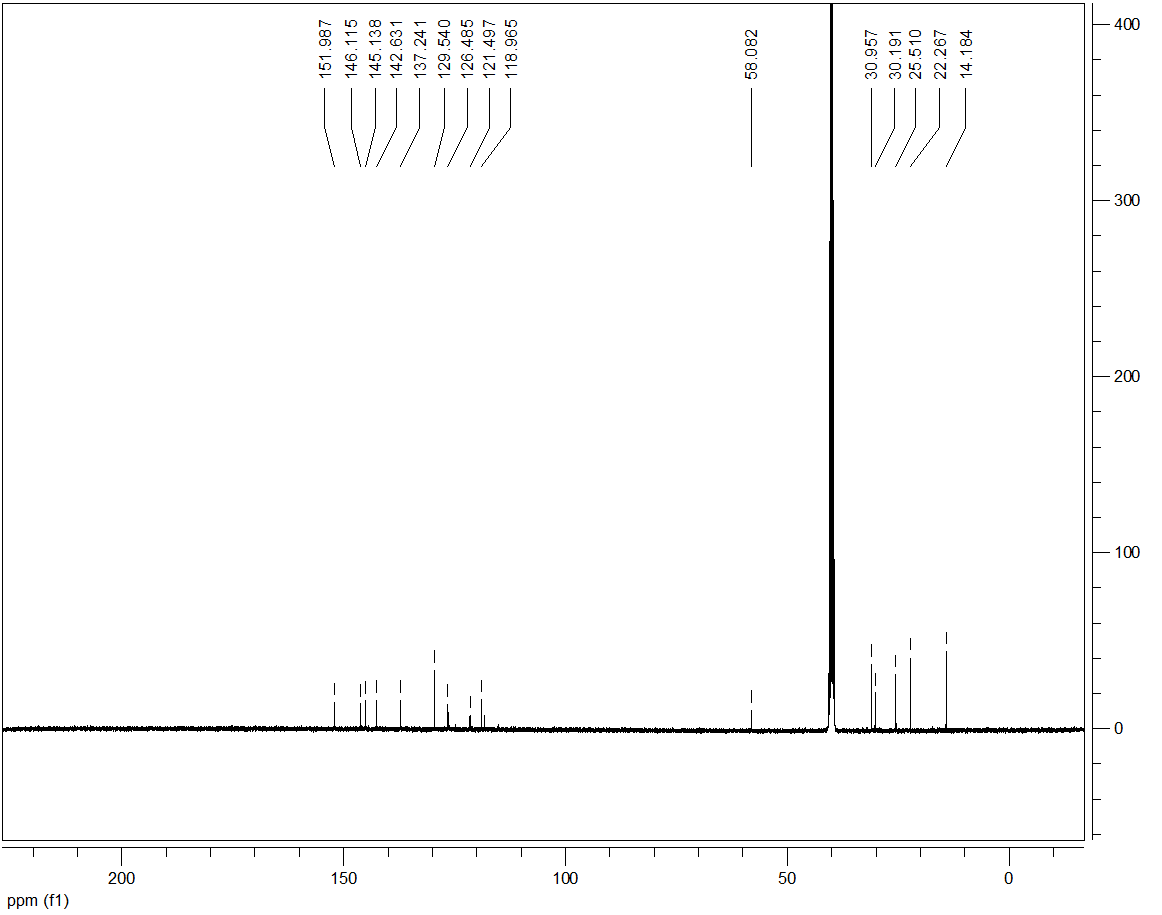


**Figure S17.** ^1^H and ^13^C NMR spectra of **2aTf_2_N** in *d*_6_-DMSO taken at room temperature.


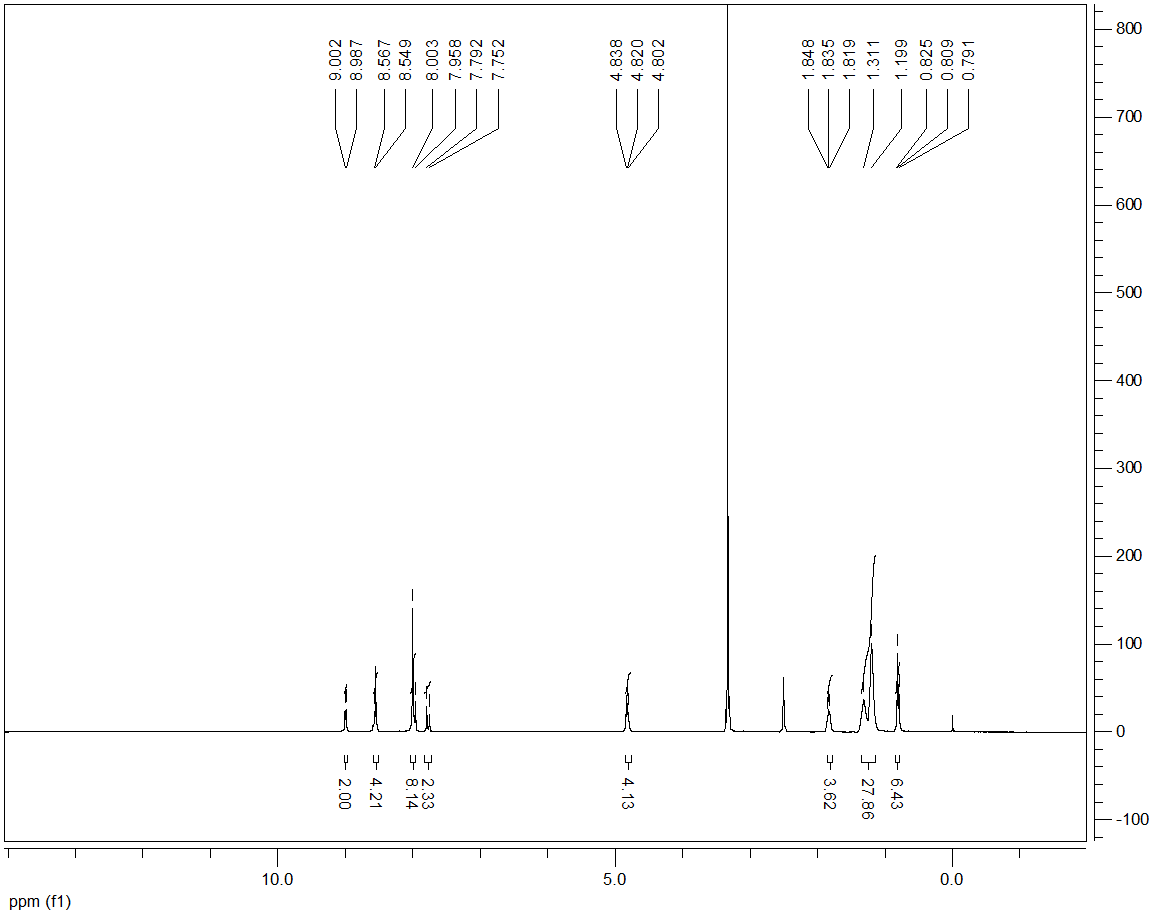


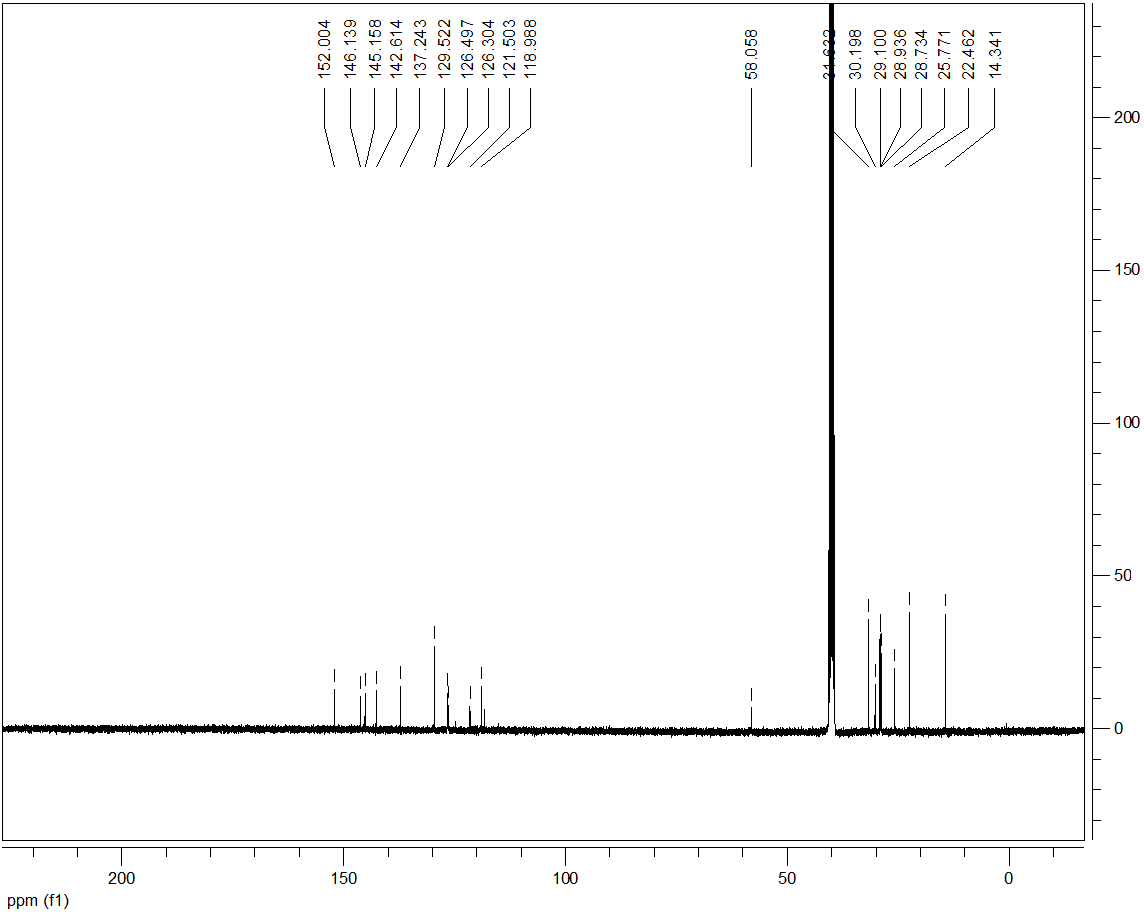


**Figure S18.** ^1^H and ^13^C NMR spectra of **2bTf_2_N** in *d*_6_-DMSO taken at room temperature.


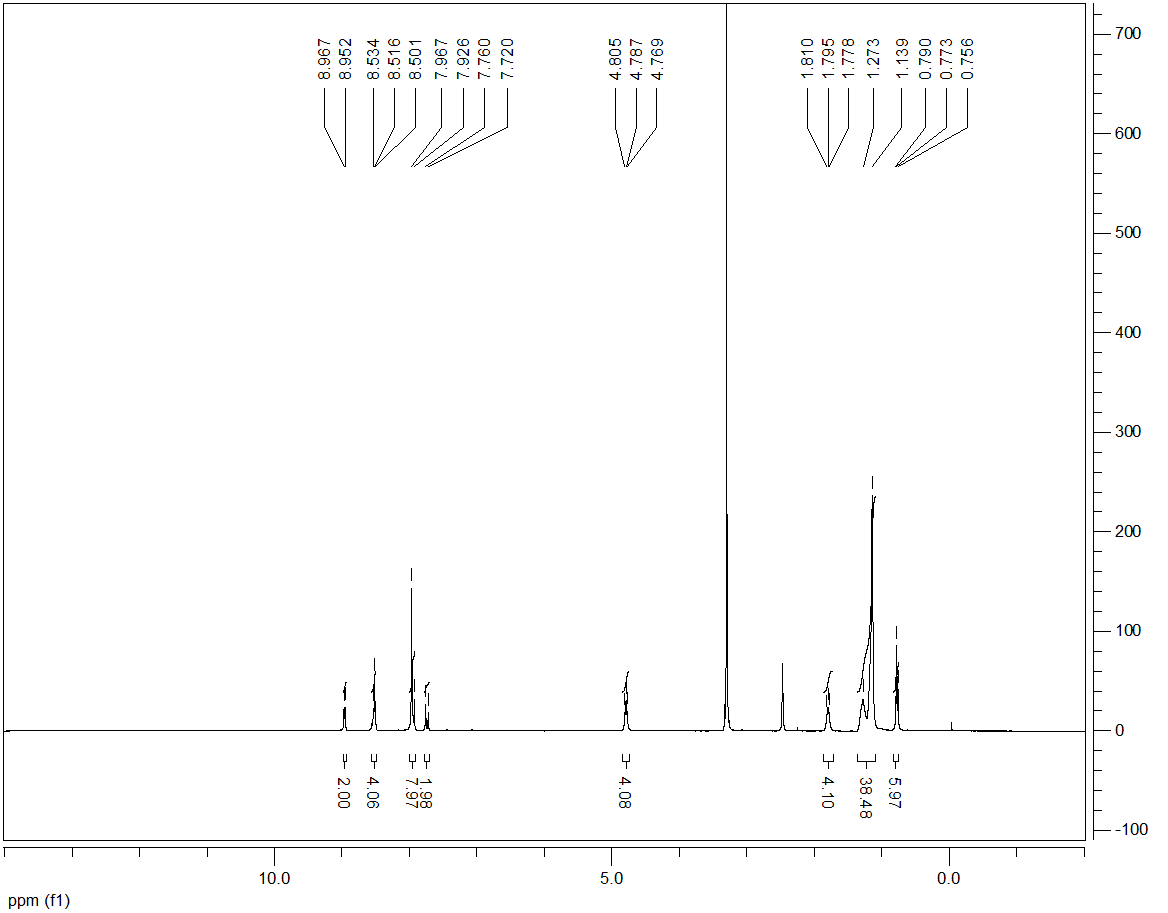


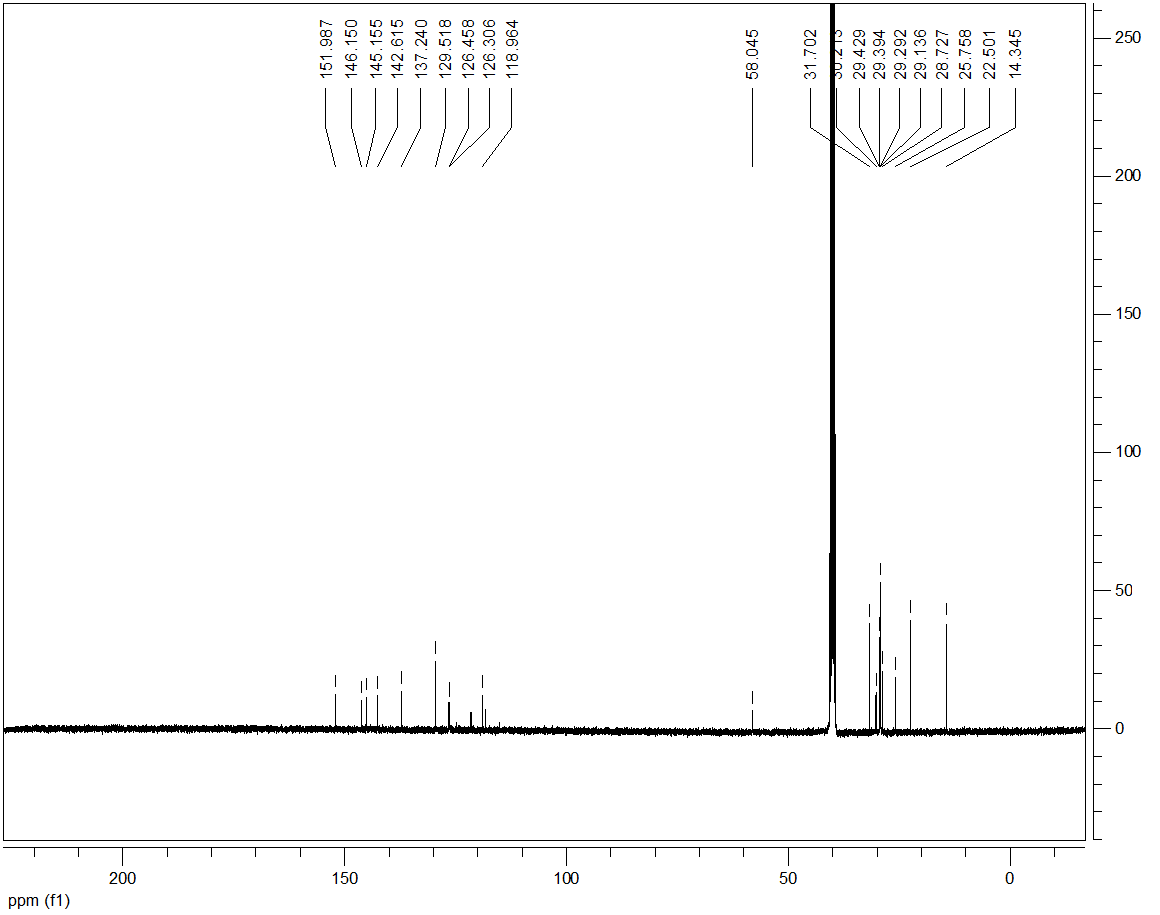


**Figure S19.** ^1^H and ^13^C NMR spectra of **2cTf_2_N** in *d*_6_-DMSO taken at room temperature.

**Table S1.** Optical absorption and photoluminescent properties of C_9_ and C_12_ *ortho-* and *para-* Q-BPEBs

| Q-BPEB | λ_abs_  wavelength (nm) | Molar absorptivity (M^-1^ cm^-1^) | λ_em_ wavelength (nm) |
| --- | --- | --- | --- |
| **1bBr** | 251  383 | ε_251_ = 19525 ±189 ε_383_ = 41544 ±247 | 485 |
| **1bOTs** | 251  381 | ε_251_ = 21284 ±380 ε_381_ = 43909 ±400 | 481 |
| **1bTf_2_N** | 251  381 | ε_251_ = 22452 ±392 ε_381_ = 47305 ±230 | 484 |
| **2bBr** | 383 | ε_383_ = 55238 ±1294 | 447 |
| **2bOTs** | 382 | ε_382_ = 54791 ±945 | 453 |
| **2bTf_2_N** | 382 | ε_382_ = 55615 ±745 | 452 |
| **1cBr** | 251  394 | ε_251_ = 22452 ±392 ε_394_ = 58781 ±2187 | 480 |
| **1cOTs** | 250  395 | ε_250_ = 20090 ±503 ε_395_ = 56412 ±1269 | 481 |
| **1cTf_2_N** | 251  395 | ε_251_ = 20447 ±130 ε_395_ = 57336 ±591 | 484 |
| **2cBr** | 385 | ε_385_ = 58062 ±1835 | 446 |
| **2cOTs** | 383 | ε_383_ = 54625 ±1148 | 450 |
| **2cTf_2_N** | 382 | ε_382_ = 50390 ±387 | 446 |

**Figure S20.** UV-Visible absorption spectrum of **1aBr** in methanol.

**Figure S21.** UV-Visible absorption spectrum of **1aOTs** in methanol.

**Figure S22.** UV-Visible absorption spectrum of **1aTf_2_N** in methanol.

**Figure S23.** UV-Visible absorption spectrum of **2aBr** in methanol.

**Figure S24.** UV-Visible absorption spectrum of **2aOTs** in methanol.

**Figure S25.** UV-Visible absorption spectrum of **2aTf_2_N** in methanol.

**Figure S26.** UV-Visible absorption spectrum of **1bBr** in methanol.

**Figure S27.** UV-Visible absorption spectrum of **1bOTs** in methanol.

**Figure S28.** UV-Visible absorption spectrum of **1bTf_2_N** in methanol.

**Figure S29.** UV-Visible absorption spectrum of **2bBr** in methanol.

**Figure S30.** UV-Visible absorption spectrum of **2bOTs** in methanol.

**Figure S31.** UV-Visible absorption spectrum of **2bTf_2_N** in methanol.

**Figure S32.** UV-Visible absorption spectrum of **1cBr** in methanol.

**Figure S33.** UV-Visible absorption spectrum of **1cOTs** in methanol.

**Figure S34.** UV-Visible absorption spectrum of **1cTf_2_N** in methanol.

**Figure S35.** UV-Visible absorption spectrum of **2cBr** in methanol.

**Figure S36.** UV-Visible absorption spectrum of **2cOTs** in methanol.

**Figure S37.** UV-Visible absorption spectrum of **2cTf_2_N** in methanol.

**Figure S38.** Emission/Excitation spectrum of **1bBr** in methanol (5.0 × 10^-5^ M).

**Figure S39.** Emission/Excitation spectrum of **1bOTs** in methanol (5.0 × 10^-5^ M).

**Figure S40.** Emission/Excitation spectrum of **1bTf_2_N** in methanol (5.0 × 10^-5^ M).

**Figure S41.** Emission/Excitation spectrum of **2bBr** in methanol (5.0 × 10^-5^ M).

**Figure S42.** Emission/Excitation spectrum of **2bOTs** in methanol (5.0 × 10^-5^ M).

**Figure S43.** Emission/Excitation spectrum of **2bTf_2_N** in methanol (5.0 × 10^-5^ M).

**Figure S44.** Emission/Excitation spectrum of **1cBr** in methanol (5.0 × 10^-5^ M).

**Figure S45.** Emission/Excitation spectrum of **1cOTs** in methanol (5.0 × 10^-5^ M).

**Figure S46.** Emission/Excitation spectrum of **1cTf_2_N** in methanol (5.0 × 10^-5^ M).

**Figure S47.** Emission/Excitation spectrum of **2cBr** in methanol (5.0 × 10^-5^ M).

**Figure S48.** Emission/Excitation spectrum of **2cOTs** in methanol (5.0 × 10^-5^ M).

**Figure S49.** Emission/Excitation spectrum of **2cTf_2_N** in methanol (5.0 × 10^-5^ M).
